# Targeting kinases with anilinopyrimidines: discovery of *N*-phenyl-*N*’-[4-(pyrimidin-4-ylamino)phenyl]urea derivatives as selective inhibitors of class III receptor tyrosine kinase subfamily

**DOI:** 10.1038/srep16750

**Published:** 2015-11-16

**Authors:** Valentina Gandin, Alessandro Ferrarese, Martina Dalla Via, Cristina Marzano, Adriana Chilin, Giovanni Marzaro

**Affiliations:** 1 Department of Pharmaceutical and Pharmacological Sciences, University of Padova, via Marzolo, I-35131, Padova (Italy)

## Abstract

Kinase inhibitors are attractive drugs/drug candidates for the treatment of cancer. The most recent literature has highlighted the importance of multi target kinase inhibitors, although a correct balance between specificity and non-specificity is required. In this view, the discovery of multi-tyrosine kinase inhibitors with subfamily selectivity is a challenging goal. Herein we present the synthesis and the preliminary kinase profiling of a set of novel 4-anilinopyrimidines. Among the synthesized compounds, the *N*-phenyl-*N*’-[4-(pyrimidin-4-ylamino)phenyl]urea derivatives selectively targeted some members of class III receptor tyrosine kinase family. Starting from the structure of *hit compound* **19** we synthesized a further compound with an improved affinity toward the class III receptor tyrosine kinase members and endowed with a promising antitumor activity both *in vitro* and *in vivo* in a murine solid tumor model. Molecular modeling simulations were used in order to rationalize the behavior of the title compounds.

Protein kinases (PKs) are key enzymes that regulate almost all cell processes. PKs transfer a phosphate group from ATP (or GTP) to specific residues (mainly tyrosine, threonine or serine) located in the target proteins, thus promoting specific pathways^1^,^2^. The human kinome contains 518 different protein kinases grouped into eight major families on the basis of structural similarity^2^,^3^. The overexpression or deregulation of kinases (in particular tyrosine kinases, TKs) are often linked to cancer onset and progression^4^. Hence, TKs are among the major classes of biological targets for modern cancer therapy^5^. The TK family contains both receptor (RTK) and cytoplasmic enzymes^3^. Among the RTKs, of particular interest are the ErbB family^6^ (or class I RKT, comprising the four receptors ErbB1-4) and the platelet-derived growth factor family^7^ (PDGF; or class III RTK). Activating mutations of ErbB1 (also known as epidermal growth factor receptor, EGFR) are correlated with onset and progression of different solid cancers^8^ and in particular with lung cancer^9^. Mutations in class III RTK members (*i.e.* Fms-like TK-3, FLT3; colony stimulating factor-1 receptor, CSF1R; KIT; PDGFRα; PDGFRβ) are associated with hyper-proliferative pathologies as hematologic neoplasms^10^,^11^, lung cancer^12^ and pancreatic cancer^13^,^14^.

The high number of FDA approved drugs in the last 15 years reveals the growing interest in TK inhibitors (TKIs)^15^,^16^. In most cases, TKIs act as ATP-mimic compounds, although several allosteric inhibitors are under investigations^17^. Cancer is a multi factorial disease and recent findings have highlighted the importance of multi targeting compounds, *i.e.* compounds able to inhibit, with comparable potencies, more than one TK^18^. However, both selective (*e.g.* erlotinib and gefitinib) and unselective (*e.g.* sunitinib and dasatinib) kinase inhibitors are useful anticancer drugs. Hence, whether multi-kinase inhibitors have significant advantages than single kinase inhibitors is still debated^19^. Only some specific kinases should be targeted by multi-inhibitors to guarantee high efficacy while maintaining an acceptable safety: in 2010 Morphy used the term “selectively nonselective” TKIs to describe compounds with an ideal profile of kinase inhibition^20^.

Currently, a number of multi kinase inhibitors have been discovered, both by chance and by design^19^. The rational design of “selectively nonselective” TKIs is a challenging and fascinating goal: the ATP binding pocket is quite conserved in the entire kinome, and it is particularly conserved inside each PKs subfamily. In this view, an achievable and promising aim could be the design/development of subfamily selective kinase inhibitors. However, as recently reported, the inhibition of all the members of a single subfamily can lead to substantial toxicity^21^.

The ATP pocket is delimited by the hinge region (containing also the gatekeeper residue), the P-loop, the C-helix and the activation loop (containing the highly conserved DFG motif; Fig. 1a). The ATP binding pocket is constituted by the adenine pocket, the hydrophilic ribose pocket and two hydrophobic regions (Fig. 1b,c). Accordingly, TKIs are commonly constituted by *i*) a nitrogen containing heterocycle able to form an H-bond with the hinge region; *ii*) an hydrophobic moiety interacting with the hydrophobic region I of the kinase; *iii*) a spacer between the heterocycle and the hydrophobic moiety (Fig. 1d,e)^15^,^22^.

A recurring motif in kinase inhibitors is the pyrimidine nucleus. Some symmetric 4,6-dianilinopyrimidines were reported as selective EGFR inhibitors^23^. However, the selectivity profile was reported only for few compounds^23^. Similar compounds were also patented by Avila therapeutics (WO2009/051822), but few kinases were considered as targets. A number of 4-anilino-6-phenylpyrimidines were patented as cyclin-dependent kinase (CDK) inhibitors (see for example WO2008/129080 and WO2011/077171). Phosphorous containing pyrimidines as CDK inhibitors were recently published^24^. Examples of FDA approved pyrimidine based TKIs are imatinib^25^ (Abl/KIT/PDGFRβ inhibitor) and dasatinib^26^ (dual Abl/Src inhibitor). Examples of compounds involved in clinical trials are BAY1000394^27^ and the fused pyrimidine PHA-848125^28^ (pan CDK inhibitors). Nevertheless, in most cases the pyrimidine nucleus is not the main “nitrogen containing heterocycle”, since it does not interact with the hinge region, as shown by X-ray crystallography^29^.

Another interesting class of TKIs is constituted by the 4-anilinoquinazolines^30^. These compounds are mainly known as high selective EGFR^31^, dual EGFR/ErbB2^32^ or dual EGFR/VEGFR2^33^ (vascular endothelial growth factor receptor-2). We have recently reported that the *m*-biphenylamine as aniline moiety led to multi kinase inhibitors targeting EGFR, fibroblast growth factor receptor-1 (FGFR1), VEGFR2, PDGFRβ, Src and Abl at nanomolar concentrations^34^,^35^. Indeed, 4-anilinoquinazolines could be considered as fused pyrimidine (*i.e.* 4-anilinobenzopyrimidine).

On these basis, we planned the synthesis of novel potential TKIs bearing the 4-anilinopyrimidine core as the nitrogen containing heterocycle (Fig. 1f). The compounds were further characterized by 5-phenyl, 6-phenyl or 6-phenylamino moieties designed to interact with the hydrophobic region II of the ATP pocket, the ribose pocket or the DFG motif (Fig. 1g). The 4-anilino portion of the compounds was supposed to interact with the hydrophobic pocket I (HP-I) of the kinase, in analogy to 4-anilinoquinazolines. The interactions with HP-I have often been used to obtain selectivity in kinase inhibitions^36^,^37^,^38^. For example, very small differences in position or nature of 4-anilinosubstituent in quinazoline compounds led to selective EGFR^39^, VEGFR2^40^ or janus kinase (JAK)^41^ inhibitors. Hence, we functionalized the 4-anilino moiety with substituent differing in the hydrophobicity/hydrophilicity, in the ability to act as H-bond donor or acceptor, in the position and in the size. Since our purpose was to determine how the functionalization of the pyrimidine nucleus at 4 and 5 or at 4 and 6 positions could modulate the kinase selectivity profile, at this stage, we considered only simple commercially available or easily accessible anilines. Several papers and patents describing very similar compounds as kinase inhibitors are present in literature (a selection of representative bibliography is reported in Table S2). The novelty of our work relies in the evaluation of a library of simple 4-anilinopyrimidines against a wide panel of kinases and human cancer cell lines to discover novel “selective unselective” hit compounds. Indeed, through this approach we identified the 6-phenyl-4-anilinopyrimidine derivative **19** as a selective dual KIT/PDGFRβ inhibitor. Compound **19** was further improved, leading to a novel and more potent KIT/PDGFRα/PDGFRβ inhibitor (**27**) endowed with a promising anticancer potential. Molecular modeling studies were used to clarify the structure activity relationships emerged by K_d_ determination. Since both **19** and **27** targeted only few specific members of a TK family, they can be considered as novel “selectively nonselective” TKIs.

## Results

### Compounds synthesis

The structures of synthesized compounds are reported in Fig. 2 (see also Table S1).

Compounds **1–7** and **8–12** were obtained starting from 4,6-dichloropyrimidine **28** (Fig. 3a). In case of unsymmetrical compounds (**1–7**), compound **28** was first condensed with a slight excess of aniline in *i*-PrOH in the presence of triethylamine (TEA) and then with the appropriate aniline derivatives in *i*-PrOH, in both cases taking advantage from the Microwave Assisted Organic Synthesis (MAOS). Diarylurea derivative **7** was obtained by the reaction of aminoderivative **6** with phenyl isocyanate in dichloromethane.

In case of symmetrical compounds (**8–12**), compound **28** was reacted with a double amount of the appropriate aniline derivatives, but in this case, the nucleophilic aromatic substitutions were performed in *i*-PrOH at reflux without the presence of TEA.

Compounds **13–19** were synthesized starting from ethyl benzoylacetate (**30**; Fig. 3b) which was reacted with thiourea in anhydrous alkaline medium obtaining the corresponding thiopyrimidinol **31**. The thiol function was removed through Ni-Raney reduction. Compound **32** was then activated toward nucleophilic substitution by reaction with POCl_3_/TEA. Finally, the chloropyrimidine **33** was condensed with the appropriate aniline derivatives in *i*-PrOH, under microwave irradiation. Compound **19** was obtained from aminoderivative **18** as above described for compound **7**.

Compounds **20–26** were synthesized adapting a previously reported synthetic strategy^42^ (Fig. 3c). Thiourea **34** was firstly condensed with *N,N*-dimethylformamide dimethyl acetal and then treated with iodomethane in order to elicit the nucleophilic properties of the sulfur atom. Intermediate **36** was then reacted with phenylacetyl chloride in the presence of TEA. In this case we did not remove the sulfur function at this stage. In fact, the process worked better first activating compound **37** toward the nucleophilic aromatic substitution, then reacting compound **38** with the appropriate anilines and finally removing the sulfur function with the Ni-Raney system. Compound **26** was obtained from aminoderivative **25** as above described for compound **7**.

Among all the synthesized compounds, only two were previously reported: compound **5** was cited as synthetic intermediate^43^, whereas **8** was patented by Zeneca (US5880130). However, the two compounds were not previously screened against a panel of kinases. Compound **19** was commercially available in the Ambinter screening library. However, as reported in the Ambinter web site, it might be available only under re-synthesis with an extended delay, thus we synthesized it by ourselves. Besides, the compound was not present in academic accessible databases (*e.g.* PubChem, Reaxys).

### Tyrosine kinases screening

Synthesized compounds were screened against a panel of 48 kinases selected within the ScanEDGE subset of DiscoverX (http://www.discoverx.com/services/drug-discovery-development-services/kinase-profiling/kinomescan/scanedge). The ScanEDGE includes 97 kinases and is an economical approach to assess compounds selectivity throughout the human kinome. All the wt-TKs along with some relevant mutants of Abl1 (T315I) and of EGFR (L858R and L858R/T790M) were chosen. The remaining 18 targets (3 Tyrosine Kinases-Like, TKLs; 15 Serine-Threonine Kinases, STKs) were selected among the remaining kinases in the ScanEDGE subset on the basis of their well established role in cancer (see Fig. S2 for a graphical representation of the selected kinases with reference to the kinome tree. Some references highlighting the role of each kinase in cancer are also reported in Table S3).

The compounds were evaluated using the KinomeScan™ platform (http://www.discoverx.com/services/drug-discovery-development-services/kinase-profiling/kinomescan), that measures the ability of the test compound to disrupt the complex between a high affinity ATP-mimic probe immobilized on a solid support and the kinase of interest. The KinomeScan is a very helpful technology for the fast and reliable screening of a number of compounds against a wide and customizable panel of kinases. Besides, it is not based on the use of hazardous radioactive ^32^P-ATP and has a very low rate of false positive (<1%)^44^. The screening platform outputs a “Percent of Control” (POC) value, statistically correlated to the dissociation constant (K_d_) value (http://www.discoverx.com/tools-resources/leadhunter-study-reports-data-analysis), for each pair of kinase/ligand. The POC is calculated as reported in equation (1):


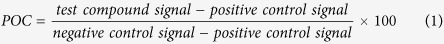


Where: test compound signal = amount of kinase still binding the probe after treatment with test compound; negative control signal = amount of kinase still binding the probe after treatment with DMSO (100% control); positive control signal = amount of kinase still binding the probe after treatment with control compound (0% control). Hence, low POC values indicate high affinity.

The compounds were tested at high concentration (10 μM), in order to highlight not only the main target but also the off-targets. The results of the preliminary screening are reported as heat map (Fig. 4; see also Table S4 for all the measured values), in which the compounds are grouped on the basis of the 6 or 5 substituent at the pyrimidine ring.

All the tested compounds were almost inactive against both TKL and STK members. The 6-phenylamino compounds targeted the ErbB family (comprising the wt-EGFR, ErbB2 and the two EGFR mutants L858R and L858R/T790M). Similarly to what previously reported^23^, these derivatives were mainly active against wt-EGFR, although with different potencies. Some 6-phenylamino compounds and the three phenylurea derivatives (**7**, **19** and **26**) were active against the members of the class III RTKs family.

### Structure activity relationship

Overall, 4-anilinopyrimidines targeted mainly the ErbB and the class III RTKs subfamilies.

With respect of targeting EGFR, the presence of a 5-phenyl or a 6-phenyl ring was detrimental (compare **1** with **13** and **20**; Fig. 5a). Hence, only 6-phenylamino-4-anilinopyrimidines were active against EGFR. For these compounds, the substitution of the 4-aniline moiety with small *meta* lipophilic substituents was preferred (*e.g.* potencies: **1**_*m*−Me_ > **4**_*p*−Me_; **8**_*m*−Me_ = **1**_*m*−Me_ = **9**_*m*−Br_ > **11**_*p*−Cl_). Conversely to what previously reported for 4-anilinoquinazolines^34^, a biphenylamino moiety was detrimental (see **2** and **10**). The L858R mutation (that causes an enhancement of the kinase activity without directly modifying the ATP site^45^) did not affect the ligand binding, whereas the T790M gatekeeper mutation dramatically impaired the interaction. Despite the high degree of similarity between wt-EGFR and ErbB2, compounds were not active against the latter kinase. The presence of small *meta* hydrophilic function (*i.e.* **3**_m−OH_) was well tolerated, still leading to selective EGFR inhibitors. Interestingly, small *para* hydrophilic substituents (OH, NH_2_) led to less selective compounds: derivatives **5** and **6** targeted EGFR, some members of the class III RTKs and AXL. The lowest selectivity was obtained when the pyrimidine core was functionalized at both 4 and 6 position with *para*-hydroxyaniline moieties: indeed compound **12** targeted a number of kinases, comprising also the T790M mutant of EGFR. We are planning further investigation on this compound. When the 4-anilino was functionalized with bulky hydrophilic function (*i.e.* a phenylurea, see compound **7**), very poor activity against EGFR was observed.

With respect of targeting class III RTKs, as above mentioned, the presence of a *para* H-bonding substituent in the 4-aniline moiety was required (see compounds **5** and **6**). When the 4-aniline moiety was functionalized with a *para*-phenylurea substituent, selective class III RTK members inhibition was obtained. Indeed, in the case of phenylurea compounds (**7**, **19** and **26**), the functionalization at 5 or 6 position of the pyrimidine nucleus played a key role in determining the degree of selectivity within the subfamily. The inhibition of KIT and PDGFRβ was not affected by pyrimidine substitution at 5 or 6 position (potencies: **7**_6−Phenylamino_ = **19**_6−Phenyl_ = **26**_5−Phenyl_). Conversely, the inhibition of PDGFRα strongly depended on both the nature and the position of the pyrimidine substituent (potencies: **26**_5−Phenyl_ > **19**_6−Phenyl_ > **7**_6−Phenylamino_). The absence of a substituent at the 6 position led to the inhibition of all the members of the subfamily (see **26**_5−Phenyl_). On the other end, the presence of the 6-phenyl gave the highest selectivity (potencies for **19**_6−Phenyl_: KIT = PDGFRβ > PDGFRα > CSF1R > FLT3). Finally, the introduction of a linker between the phenyl and the pyrimidine nuclei gave an intermediate selectivity degree (potencies for **7**_6−Phenylamino_: KIT = PDGFRβ = FLT3 > CSF1R > PDGFRα).

The main structure activity relationships are resumed in Fig. 5b.

### Cytotoxicity

Compounds active against at least one kinase among EGFR and class III RTKs were evaluated for their cytotoxic properties against two different human cancer cell lines, namely non small cells A549 lung cancer cells (sensitive to EGFR inhibitors)^46^ and pancreatic BxPC3 adenocarcinoma (sensitive to both PDGFRβ and EGFR inhibitors)^47^,^48^ cells. For comparison purposes, sunitinib (SU), a multi-kinase inhibitor^49^, was evaluated under the same experimental conditions. The results, expressed as IC_50_ values (μM) calculated from the dose-survival curves obtained after 72 h of drug treatment from the MTT test, are reported in Table 1.

The newly synthesized compounds (excluding **8**, **9** and **12**) showed a cytotoxic potency in the micromolar range against both cancer cell lines. In particular, the two phenylurea derivatives **7** and **19** (see Fig. 5a for structures) were up to about 6 times more effective than the reference TKI against both A549 and BxPC3 cells (see potency relative to SU, calculated as the ratio between IC_50_ of sunitinib and IC_50_ of tested compounds in Table 1). Tested against non-tumor cells in rapid proliferation, the human embryonic kidney HEK293 cells, **19** elicited selectivity index values (SI = quotient of the IC_50_ toward non cancerous cells divided by the average IC_50_ for the malignant cells) approximately 1.5-folds higher than that calculated with SU, thus attesting a preferential cytotoxicity versus neoplastic cells. On the contrary, compound **7** was more cytotoxic against non transformed cells (SI < 1).

### Improvement of pharmacodynamic properties of *hit compound* 19

**Figure 1:**
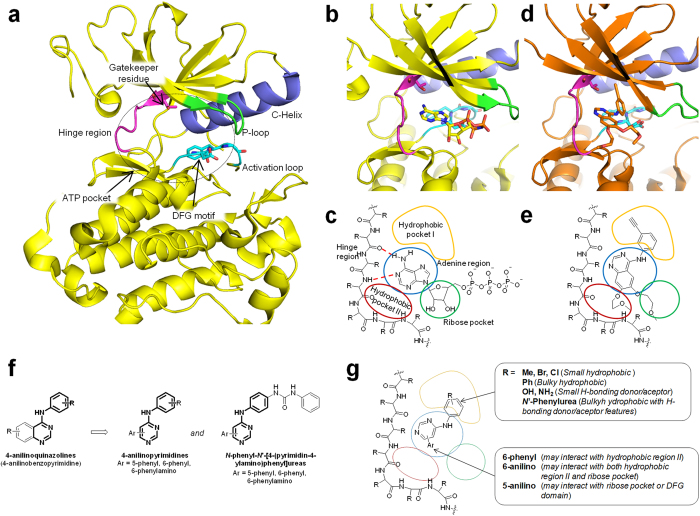
Structure of kinases and of designed compounds. (**a**) Main element in kinase structure. (**b**) Binding of AMP-ANP (PDB ID: 2GS7). (**c**) Schematic representation of binding of ATP and features of the ATP binding pocket. (**d**) Detail of binding of a kinase inhibitor (erlotinib; PDB ID: 1M17). (**e**) Schematic representation of binding of inhibitor (erlotinib) with respect to the features of the ATP binding pocket. Image partially readapted from ref. 22. (**f**) General structures of designed compounds and their structural relationship with 4-anilinoquinazolines. (**g**) Supposed binding mode of designed compounds in the ATP binding pocket.

**Figure 2:**
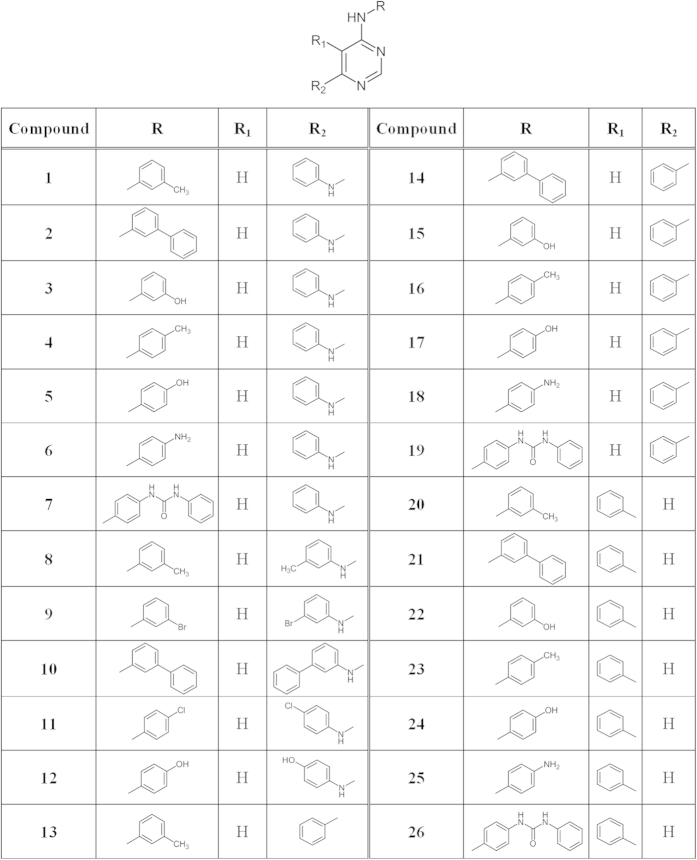
Structures of synthesized compounds.

**Figure 3:**
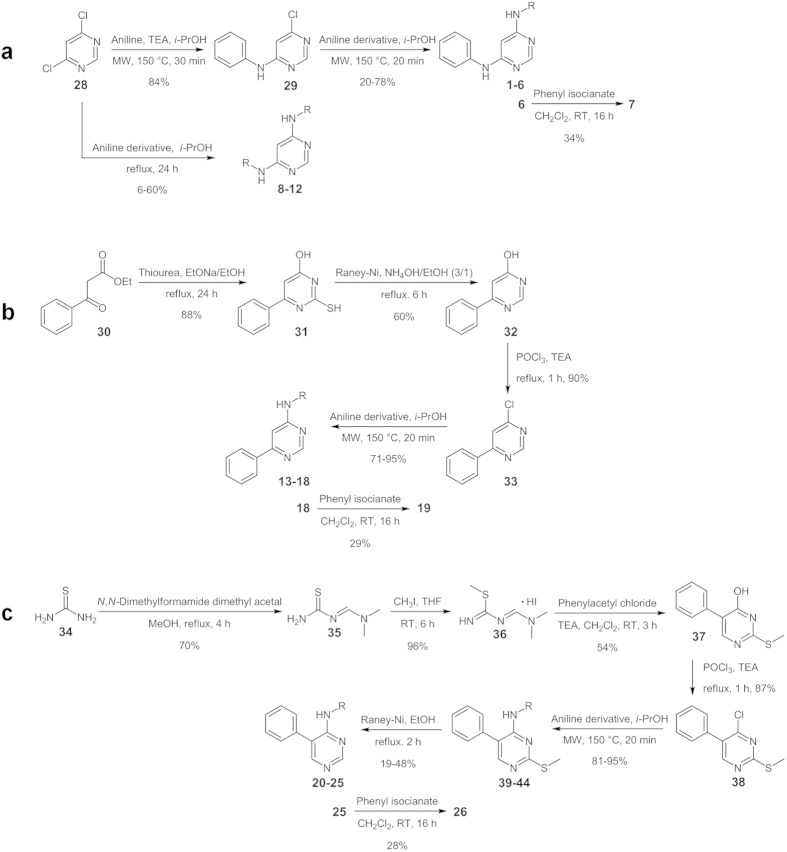
Synthesis of compounds 1–26. See Fig. 2 for R specification.

**Figure 4:**
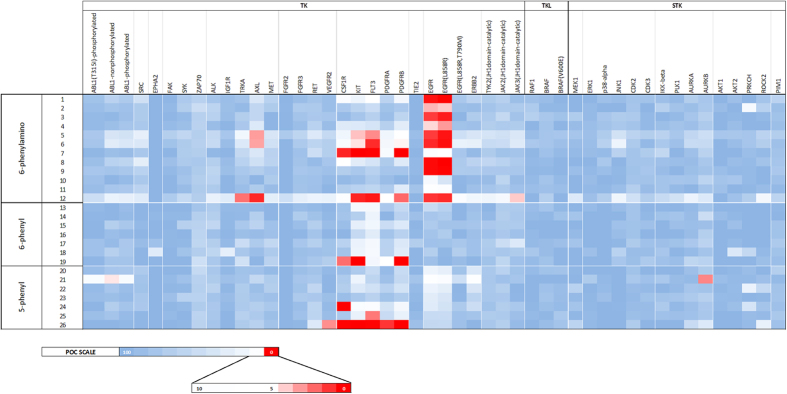
Results of primary screening of compounds against kinases. The heat map displays the results of the primary screening in terms of POC values for each pair of kinase/ligand. The lower the POC (white to red boxes), the tighter the binding. Compounds are listed according to Fig. 2.

**Figure 5:**
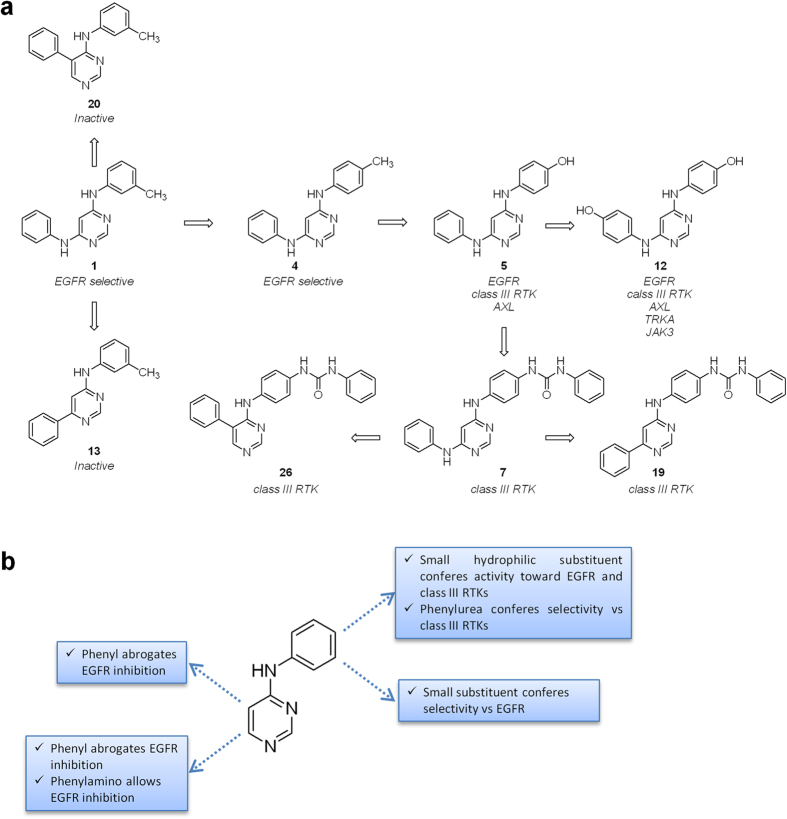
Structure activity relationships for 4-anilino compounds. (**a**) Selectivity profile of some selected compounds highlights the features required for selective EGFR, dual EGFR/class III RTKs or selective class III RTKs inhibition. (**b**) Schematic representation of main structure activity relationships with respect to 4-anilinopyrimidine core.

**Figure 6:**
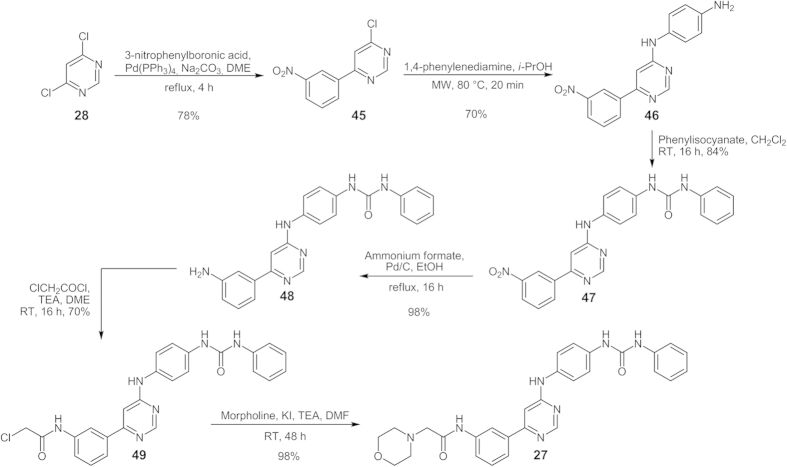
Synthesis of compound 27.

Based on the kinases screening results and on the IC_50_ and the SI values, we selected derivative **19** as our “*hit compound*”. As mentioned above, the 6-phenyl ring was designed to occupy the hydrophobic pocket II (Fig. 1g), which is next to the hydrophilic ribose binding region of the kinases (Fig. 1c). Hence, we tried to improve the ability of the hit compound to interact with class III RTK members through the insertion of a small hydrophilic moiety, intended to occupy the sugar pocket of the kinases, thus obtaining compound **27** (Fig. 6; see also Supplementary Information for details on synthetic procedures).

The ability of compounds **19** and **27** to bind the class III RTK members was measured (Table 2; see also Figs S3 and S4 for dose/response curves).

As supposed, the further functionalization of the 6-phenyl ring with a polar moiety able to interact with the sugar pocket improved the binding affinity toward all the tested kinases. Nevertheless, for both compounds the main target remained KIT. The highest affinity improvement was observed for PDGFRα (nearly an order of magnitude), whereas the interaction with PDGFRβ was not substantially modified. Compound **19** was confirmed as a dual KIT/PDGFRβ inhibitor, while compound **27** should be considered as a KIT/PDGFRα/PDGFRβ inhibitor.

The cytotoxic profile of **19** and **27** was assessed on a wider panel of human tumor cell lines including also examples of breast (MCF-7), cervical (A431), colorectal (HCT-15), and ovarian (2008) cancers as well as melanoma (A375), besides lung (A549) and pancreatic (BxPC3) cancer cells. IC_50_ values, calculated from the dose-survival curves obtained after 72 h of drug treatment from the MTT test, are reported in Table 2. Anilinopyrimidines **19** and **27** showed a quite similar pattern of cytotoxicity over the cancer cell lines panel. Both compounds were endowed with a cytotoxic potency higher than that of SU, eliciting IC_50_ values in the low micromolar range. Notably, IC_50_ values measured on pancreatic adenocarcinoma BxPC3 cells were in the sub-micromolar range, exceeding by a factor of 6 those detected with the reference TKI. When tested against non-cancer HEK293 cells, derivative **27** showed an antiproliferative activity even lower than that of the parental compound **19**, eliciting an average IC_50_ value of 10 μM, thus suggesting a preferential activity towards cancerous cells.

### Preliminary *in vivo* evaluation of lead compound 27

**Figure 7:**
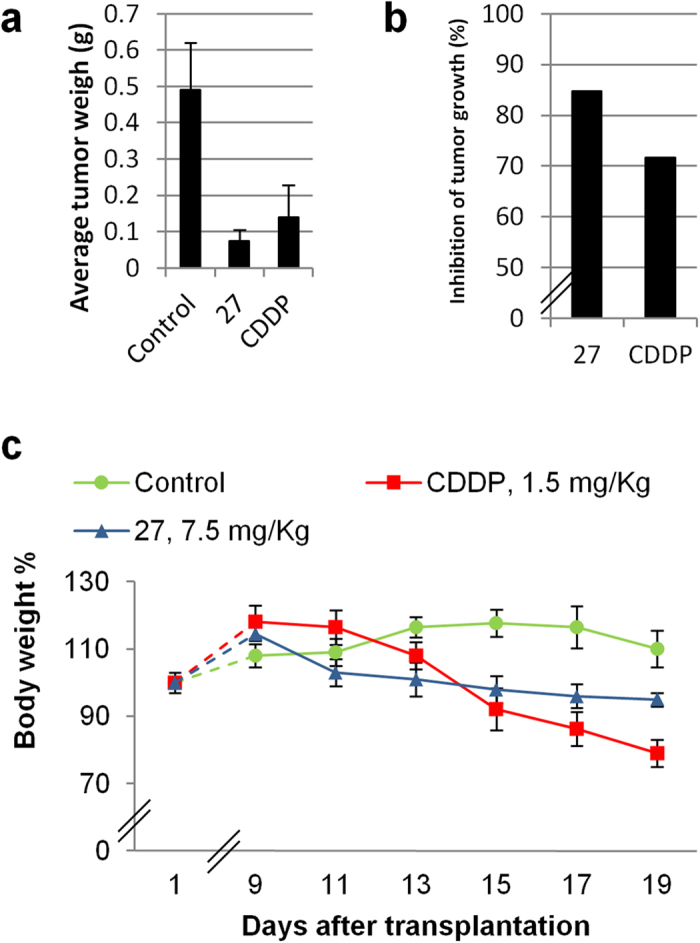
*In vivo* testing of compound 27. Lewis lung carcinoma (LLC) was implanted i.m. (2 · 10^6^ cells inoculum) into the right hind leg of 8-week old imbred C57BL mice. Nine days after tumor inoculation (palpable tumor), tumor-bearing mice were randomized into vehicle control and treatment groups (8 mice per group). Compound **27** was dosed daily at 7.5 mg/kg ip and CDDP was dosed daily at 1.5 mg/kg ip. At day 20, animals were sacrificed (*i.e.*, before tumor can cause the animal discomfort), the legs were amputated at the proximal end of the femur, and the inhibition of tumor growth was determined according to the difference in weight of the tumor-bearing leg and the healthy leg of the animals expressed as % referred to the control animals. Control was constituted by vehicle (0.2%_v/v_ EtOH and 99.8%_v/v_ of saline solution). CDDP was used as positive reference. Both **27** and CDDP were administered at the highest not toxic doses (7.5 and 1.5 mg/kg, respectively), as determined by MTD studies. (**a**) Average tumor weight (**g**) after 20 days of treatment with control or compounds. (**b**) Percentage of reduction in tumor growth after 20 days of treatment with respect to control mice. (**c**) Body weight changes. The body weight changes of LLC bearing C57BL mice treated with vehicle or tested compounds. Each drug was administered daily after 9 days from the tumor cell inoculum. Weights were measured at day 1 and daily from day 9. Error bars indicate the standard deviation.

Basing on *in vitro* screening, derivative **27** emerged as the most interesting compound since it was more effective than **19** against target kinases. Besides, it was as cytotoxic as **19** towards cancer cells while showing a more favorable selectivity index. On these basis, compound **27** was chosen for *in vivo* experiments against a syngeneic murine solid tumor model, the murine Lewis Lung Carcinoma (LLC). The tumor growth inhibition induced by **27** was compared with that promoted by cisplatin (CDDP), the most common chemotherapeutic drug used in the treatment of lung cancers. Nine days after tumor inoculation, tumor-bearing mice were randomized into vehicle control and treatment groups (8 mice per group). Control mice received the vehicle (0.2%_v/v_ EtOH and 99.8%_v/v_ of a saline solution), whereas treated groups received daily doses of **27** (7.5 mg · kg^−1^ in the vehicle solution composed of 0.2%_v/v_ EtOH and 99.8%_v/v_ of saline solution) or CDDP (1.5 mg · kg^−1^ in saline solution). Tumor growth was estimated at day 20 (Fig. 7a). As an indication of the adverse side effects, changes in the body weights of tumor-bearing mice were monitored at day 1 and daily from day 9. The intraperitoneal administration of **27** reduced by 84.7% the tumor mass compared to that of the control group (Fig. 7b, Table S5), promoting an *in vivo* antitumor activity even better than that exerted by CDDP which reduced the tumor mass of 71.6%.

Remarkably, even though **27** was administered at higher doses than CDDP, the time course of body weight changes indicated a more safety profile for our compound. Indeed, treatment with **27** resulted in a moderate body weight loss (<10%) whereas, as well documented, CDDP provoked elevated body weight loss (Fig. 7c).

### The molecular basis for class III RTKs subfamily selectivity

**Figure 8:**
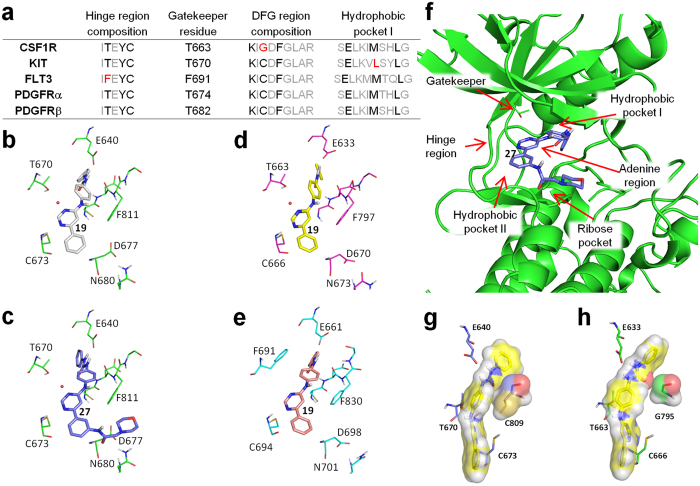
Binding mode study on compounds 19 and 27. (**a**) Sequences of hinge, DGF and hydrophobic pocket I regions of the ATP binding pocket for class III RTKs. Black (or red) characters indicate amino acids that place the side chain inside the ATP-pocket; grey characters indicate the amino acids that place the side chain outside the ATP-pocket. Amino acids that place the side chain inside the ATP-pocket and that differ between the five kinases are highlighted in red. The gatekeeper residue number is also reported. (**b**–**f**) Results of docking simulations: (**b**) Potential binding mode of **19** (gray stick) with KIT (green sticks); (**c**) Potential binding mode of **27** (blue stick) with KIT (green sticks); (**d**) Potential binding mode of **19** (yellow sticks) with CSF1R (pink sticks); (**e**) Potential binding mode of **19** (light pink sticks) with FLT3 (cyan sticks); (**f**) Potential binding mode of **27** (blue sticks) with KIT (green sticks and green cartoons), in context with the ATP binding site features. The 6-phenyl moiety of compound **27** is placed in the hydrophobic pocket II, whereas the hydrophilic function (*i.e.* the morpholine ring) is placed in the ribose pocket. (**g**,**h**) Details of docking simulations for compound **19** (yellow sticks) in KIT (blue sticks, panel (**g**)) and CSF1R (green sticks, panel (**h**)). Compound **19**, KIT_C809_ and CSF1R_G795_ are depicted as transparent surfaces. The gatekeeper residues (KIT_T670_ and CSF1R_T663_) as well as other protein residues are depicted as sticks in order to allow a better comprehension of the compound binding modes.

To rationalize the selectivity profile showed by compounds **19** and **27**, we firstly analyzed some key residues of the ATP-pocket usually involved in the binding of TKIs (Fig. 8a).

The class III RTK members mainly differ in:

1. the gatekeeper residue (threonine for CSF1R, KIT, PDGFRα and PDGFRβ; phenylalanine for FLT3). Threonine and phenylalanine don’t have similar steric requirements (blocks substitution matrix, BLOSUM^50^, score_F−T_ = −2);
2. one amino acid in the DFG region sequence (cysteine for KIT, FLT3, PDGFRα and PDGFRβ; glycine for CSF1R). Cysteine and glycine don’t have similar steric requirements (BLOSUM score_C−G_ = −3);
3. the composition of the deeper part of hydrophobic pocket I, accessible only in the inactive kinase conformation (usually targeted by type II inhibitors), that was reported to account for the selectivity of certain type II kinase inhibitors^51^. Considering only the amino acids placing the side chains inside the binding pocket, the hydrophobic pockets differed only for one amino acid (leucine in KIT; methionine in the other kinases). However, leucine and methionine have similar steric requirements (BLOSUM score_M−L_ = +2). Remarkably, the sequences *xExxxLxxL* or *xExxxMxxL* are quite conserved in the TK family (see for example Src, Syk, ZAP70, TRKA, TIE2 and TYK2), thus the composition of the deeper part of hydrophobic pocket I could not mainly account for the overall observed selectivity of the compounds.

Since both **19** and **27** targeted preferentially KIT, PDGFRα and PDGFRβ, we supposed that the simultaneous presence of the threonine as gatekeeper and of the cysteine just before the DFG motif was fundamental. Notably, no other TKs (among the tested ones) presented these two features. Hence, we concluded that the selectivity observed for some members of the class III RTK was mainly due to the presence of the two residues.

Molecular modeling studies were then conducted on both **19** and **27** in order to propose a plausible binding mode with the class III RTK members. From among the available crystal structures, we selected 1T46 for KIT^52^, 1RJB for FLT3^53^ and 4HW7 for CSF1R^54^. These structures were chosen on the basis of the similarity with 4ASD (VEGFR2 kinase domain in complex with sorafenib^55^) because of the high degree of similarity of sorafenib with our compounds. Remarkably, no structure for PDGFRα and β were available in the Protein Data Bank, thus these kinases were no further considered in computational simulations. The docking experiments were conducted using the AutoDock software^56^. The importance of the gatekeeper residue suggested the presence of a water molecule mediated H-bond between the N3 of the pyrimidine and the threonine, as previously reported for the binding of quinazoline derivatives and EGFR (see for example the 1M17 PDB structure^57^). Remarkably, the supposed water molecule was not present in the 1T46 and 4HW7 protein structures. As recently reported, AutoDock software was implemented with a specific force field with discrete displaceable waters in order to dock hydrated ligands^58^. Following this approach, water molecules were added to compounds **19** and **27** and then docked in the protein structures (Fig. 8b–e; the full details of the binding mode for **19** and **27** are reported in Figures from S5 to S8).

As supposed, a water molecule classified as “strong” by the scoring protocol^58^ mediated the binding of both **19** and **27** with the threonine gatekeeper in KIT and CSF1R kinases, whereas it was displaced in FLT3. The lack of the water mediated H-bond in FLT3 was partially replaced by an edge-to-face arene-arene interaction between the aniline moiety and the F691, justifying the lower but not negligible binding. Beside the H-bonds, an additional edge-to-face arene-arene interaction was observed for the 4-anilino moiety of both **19** and **27** and the F811 of the DFG domain of KIT (centroids distance: 5.5 Å in both cases). The binding mode predicted for **27** in KIT was consistent with the initial hypothesis since the hydrophilic moiety interacted with the residues in the ribose pocket (Fig. 8f).

We also investigated the role of cysteine of the DFG domain. The KIT_C809_ (Fig. 8g) showed higher surface complementarity than the CSF1R_G795_ (Fig. 8h) with the urea moiety of the ligand. Moreover, the distances between the sulfur atom of C809 and the aniline moiety suggested the formation of a sulfur-arene interaction^59^. Furthermore, the higher steric hindrance of C809 constrained the urea moiety in a very favorable position to interact with the KIT_E640_ carboxylic function.

## Discussion

Tyrosine kinases inhibitors are among the most attractive and promising anti-cancer compounds. Both selective and unselective kinase inhibitors have been approved for cancer treatment. For multi-kinase inhibitors a correct balance between specificity and non-specificity is required^20^. In this view, the discovery of compounds with subfamily selectivity is a challenging goal. The development of high-throughput screening technology to screen compounds against a number of kinases is having a high impact in the discovery of novel hit with interesting selectivity profile^60^. Due to our interest in discovering novel TKIs, we decided to explore the feasibility of obtaining novel multi-target TKIs bearing 4-anilinopyrimidine structure. Firstly we synthesized a small focused library of 26 different 4-anilinopyrimidine derivatives: the main scaffold was functionalized with different substituent at the 4-anilino moiety and at the 5 and 6 positions of the pyrimidine nucleus to determine the molecular features required for kinases inhibition. Then, the library was screened against a panel of 48 different kinases (comprising TKs, TKLs and STKs). All the compounds were inactive against STKs and TK-like enzymes. Among TKs, the compounds were able to target mainly EGFR and/or the member of the class III RTKs family (CSF1R, FLT3, KIT, PDGFRα and PDGFRβ). The substitution of the pyrimidine nucleus at 5 or 6 positions was fundamental: compounds bearing a 5-phenyl or a 6-phenyl moiety resulted totally inactive against EGFR. Conversely, almost all the 6-phenylamino compounds bound both wt-EGFR and L858R-EGFR mutant.

In the case of 6-phenylamino compounds, the 4-anilino moiety could be functionalized with small lipophilic substituents (methyl, bromo, chloro) at *meta* or *para* positions, with a slight preference for the *meta* position. The functionalization with a small *para* hydrophilic function (**5**_p−OH_, **6**_p−NH2_) extended the inhibitory spectrum, leading to EGFR/class III RTK/AXL inhibitors. The introduction of hydroxylic functions at both 4-anilino and 6-anilino moieties led to the wider spectrum of activity: compound **12** was able to target also TRKA and JAK3. Besides, compound **12** retained a low but not negligible activity also against the T790M mutant of EGFR. When the 4-aniline was functionalized with a *para*-phenylurea moiety, we obtained a selective class III RTKs inhibitor (**7**). Compound **7** probably was a type II inhibitor, thus requiring an inactive kinase conformation. However, EGFR is known to adopt a Src-like inactive conformation^61^, which substantially differs from the KIT inactive conformation^52^: the DFG domain of inactive EGFR still remains close to the ATP binding pocket, thus limiting the dimension of the hydrophobic pocket I and impairing the binding with compound **7**. Conversely, inactive KIT adopt a true DFG-out conformation with a larger hydrophobic pocket I.

Remarkably, all the compounds bearing the *para*-phenylurea moiety (**7**, **19** and **26**) were selectively active against the class III RTK members. In this case, the substitution at 5 or 6 positions of the pyrimidine ring conferred selectivity inside the family: 5-phenyl compound (**26**) inhibited all the class III RTK members with comparable potencies; 6-phenyl compound (**19**) was a selective dual KIT/PDGFRβ inhibitor; 6-phenylamino compound (**7**) showed an intermediate selectivity degree between **19** and **26**.

Compounds active against at least one of the main target kinases (EGFR and/or class III RTKs) were screened for their cytotoxic potential against lung and pancreatic cancer cell lines. Many of the studied compounds showed a marked *in vitro* antitumor activity, with IC_50_ values in the micromolar range. However, only **7** and **19** showed lower average IC_50_ values than the reference multi TKI, sunitinib. In addition, when tested against non-tumor cells in rapid proliferation, only compound **19** showed a preferential antiproliferative activity toward neoplastic cells.

Dissociation constant determinations confirmed that compound **19** was a selective dual KIT/PDGFRβ inhibitor with nanomolar affinity. The structure of this *hit compound* was further modified in order to improve the pharmacodynamic properties. Hence, compound **27** (designed to interact also with the sugar pocket of the target kinases) showed overall lower Kd values against class III RTK members than **19**, resulting a multi KIT/PDGFRα/PDGFRβ inhibitor with nanomolar affinity (Kd against CSF1R and FLT3 were higher than 1 μM).

Tested against a wide panel of cancer cell lines, including examples of breast, ovarian cancers along with melanoma, compounds **19** and **27** showed a comparable activity profile and were more effective than sunitinib in inhibiting cancer cell proliferation. Remarkably, compound **27** was more selective against cancer cells than the hit compound **19**. Very preliminary *in vivo* studies were also conducted on compound **27**. On LLC murine model of solid tumor, compound **27** promoted a reduction of tumor mass higher than CDDP, the most widely used drug in the management of lung neoplasms, coupling with a reduced host weight loss. Based on these promising results, we are now planning pharmacokinetic studies on compound **27** in order to determine its effective anti-cancer.

Molecular modeling studies were conducted on compounds **19** and **27** to investigate the molecular basis for the class III RTKs subfamily selectivity. The threonine as gatekeeper and the presence of a cysteine residue in the DFG domain played a key role in determining the selectivity profile. On the contrary, the hydrophobic pocket I composition was not fundamental for the selectivity profile. These findings suggest that in the design of “selectively unselective” TKIs the gatekeeper and the DFG domain compositions must be strongly considered. In particular, the presence of sulfur-arene interactions (for CDFG containing kinases) and the role of water mediated hydrogen bonds (for threonine gatekeeper containing kinases) may play critical roles. Furthermore, as supposed, docking studies suggested that the 6-phenyl moiety of compound **27** interacted with both the hydrophobic pocket II and with the sugar pocket of the kinases.

Overall, our data suggested that the *N*-phenyl-*N*’-[4-(6-phenylpyrimidin-4-ylamino)phenyl]urea derivatives constitute a promising class of subfamily selective inhibitors of class III RTKs deserving further development.

## Methods

### Computational Methodologies

All the computational studies were carried out on a 4 CPU (Intel Core2 Quad CPU Q9550, 2.83 GHz) ACPI ×64 Linux workstation with Ubuntu 12.04 operating system. The tridimensional structure of the kinases were downloaded from Protein Data Bank (PDB ID: 4HW7 for CSF1R in complex with PLX647-OME; 1T46 for KIT in complex with imatinib; 1RJB for FLT3). The structures were superimposed using the UCSF Chimera software^62^ and the ligands, the ions and the water molecules were removed. The structure of compounds **19** and **27** were prepared with MarvinSketch 5.5.0.1 software (www.chemaxon.com/products). The lowest energy conformations and the degree of protonation at pH 7.4 were determined with OpenBabel software^63^ using the MMFF94s force field. For all the molecules, the appropriate “.pdbqt” files were prepared with the AutoDockTools graphical interface of AutoDock 4 software^56^. Before docking simulations, the “.pdbqt” file of compounds **19** and **27** were solvated using the appropriate python script^33^ (more details are reported in Supplementary Information). All the docking studies have been performed with AutoDock4 using a docking box of 52 × 70 × 40 Å dimensions (centered on PLX647-OME coordinates) and 0.375 grid spacing. Each docking run consisted of 50 independent searches with a maximum of 2,500,000 energy evaluations based on Lamarkian Genetic Algorithm searching engine. For each pair kinase/ligand the lowest energy pose was retained.

### Experiments with Cultured Human Cells

All compounds, except derivative **27**, were dissolved in DMSO just before the experiment, and a calculated amount of drug solution was added to the cell growth medium to a final solvent concentration of 0.5%, which had no discernible effect on cell killing. Compound **27** was dissolved in a 0.9% NaCl solution just before the experiment.

Sunitinib malate and MTT (3-(4,5-dimethylthiazol-2-yl)-2,5-diphenyltetrazolium bromide) were obtained from Sigma Chemical Co, St.Louis, USA. Antibody for β-actin and ubiquitin were from Santa Cruz Biotechnology (Santa Cruz Biotechnology Inc., Santa Cruz, CA, USA).

### Cell cultures

Human lung (A549), breast (MCF-7), pancreas (BxPC3), and colon (LoVo) carcinoma cell lines along with melanoma (A375) were obtained from American Type Culture Collection (ATCC, Rockville, MD). Human non-tumor embryonic kidney HEK293 cells were obtained from European Collection of Cell Cultures (ECACC, Salisbury, UK). Human cervical carcinoma A431cells were kindly provided by Prof. F. Zunino (Division of Experimental Oncology B, Istituto Nazionale dei Tumori, Milan, Italy). Human ovarian cancer 2008 cells were kindly provided by Prof. G. Marverti (Dept. of Biomedical Science of Modena University, Italy). Cell lines were maintained in the logarithmic phase at 37 °C in a 5% carbon dioxide atmosphere using the following culture media containing 10% fetal calf serum (Euroclone, Milan, Italy), antibiotics (50 units ∙ mL^−1^ penicillin and 50 μg ∙ mL^−1^ streptomycin) and 2 mM l-glutamine: i) RPMI-1640 medium (Euroclone) for MCF-7, A431, BxPC3 and 2008 cells; ii) F-12 HAM’S (Sigma Chemical Co.) for A549 and LoVo cells; iii) D-MEM medium (Euroclone) for HEK293 cells.

### Cytotoxicity MTT assay

The growth inhibitory effect towards human cell lines was evaluated by means of MTT (tetrazolium salt reduction) assay. Briefly, 3–8 ∙ 10^3^ cells/well, dependent upon the growth characteristics of the cell line, were seeded in 96-well microplates in growth medium (100 μL) and then incubated at 37 °C in a 5% carbon dioxide atmosphere. After 24 h, the medium was removed and replaced with a fresh one containing the test compound at the appropriate concentration. Triplicate cultures were established for each treatment. After 72 h, each well was treated with 10 μL of a 5 mg ∙ mL-1 MTT (3-(4,5-dimethylthiazol-2-yl)-2,5-diphenyltetrazolium bromide) saline solution, and after 5 h additional incubation, 100 μL of a sodium dodecylsulfate (SDS) solution in HCl 0.01 M were added. After overnight incubation, the inhibition of cell growth induced by the tested complexes was detected by measuring the absorbance of each well at 570 nm using a Bio-Rad 680 microplate reader (Bio-Rad, Hercules, CA). Mean absorbance for each drug dose was expressed as a percentage of the control untreated well absorbance and plotted *vs* drug concentration. IC_50_ values represent the drug concentrations that reduced the mean absorbance at 570 nm to 50% of those in the untreated control wells.

### *In vivo* anticancer activity toward Lewis Lung Carcinoma (LLC)

All studies involving animal testing were carried out in accordance with the ethical guidelines for animal research adopted by the University of Padua, acknowledging the Italian regulation (D.L.G.S. 116/92) and European Directive 86/609/EEC as to the animal welfare and protection and the related codes of practice. The experimental protocol was approved by the Italian Health Department according to the art. 7 of above mentioned D.L.G.S. 116/92. The mice were purchased from Charles River, Italy, housed in steel cages under controlled environmental conditions (constant temperature, humidity, and 12 h dark/light cycle), and alimented with commercial standard feed and tap water ad libitum. The LLC cell line was purchased from ECACC, United Kingdom. The LLC cell line was maintained in DMEM (Euroclone) supplemented with 10% heat inactivated fetal bovine serum (Euroclone), 10 mM L-glutamine, 100 U mL^−1^ penicillin, and 100 μg · mL^−1^ streptomycin in a 5% CO_2_ air incubator at 37 °C. The LLC was implanted intramuscularly (i.m.) as a 2 × 10^6^ cell inoculum into the right hind leg of 8 week old male and female C57BL mice (24 ± 3 g body weight). After 9 days from tumor implantation (palpable tumor), mice were randomly divided into 3 groups (8 animals per group) and subjected to daily i.p. administration of **27** (7.5 mg · kg^−1^ dissolved in a vehicle solution composed of 0.2%_v/v_ EtOH and 99.8%_v/v_ of saline solution), cisplatin (1.5 mg · kg^−1^ in saline solution), or the vehicle solution (0.2%_v/v_ EtOH and 99.8%_v/v_ of saline solution). At day 20, animals were sacrificed, the legs were amputated at the proximal end of the femur, and the inhibition of tumor growth was determined according to the difference in weight of the tumor-bearing leg and the healthy leg of the animals expressed as a percentage referring to the control animals. Body weight was daily measured and was taken as a parameter for systemic toxicity. All reported values are the means ± SD of no less than three measurements. Multiple comparisons were made by the Tukey−Kramer test (**p < 0.01; *or °p < 0.05).

### Statistical analysis

All the values are the means ± S.D. of not less than three measurements. Multiple comparisons were made by ANOVA followed by Tukey–Kramer multiple comparison test, using GraphPad Software.

## Additional Information

**How to cite this article**: Gandin, V. *et al.* Targeting kinases with anilinopyrimidines: discovery of *N*-phenyl-*N*′-[4-(pyrimidin-4-ylamino)phenyl]urea derivatives as selective inhibitors of class III receptor tyrosine kinase subfamily. *Sci. Rep.* **5**, 16750; doi: 10.1038/srep16750 (2015).

## Supplementary Material

Supplementary Information

## Figures and Tables

**Table 1:** Cytotoxicity of selected compounds against A549, BxPC3 and HEK293 cell lines.

ID	A549 IC_50_ (μM) ± SD	Potency relative to SU	BxPC3 IC_50_ (μM) ± SD	Potency relative to SU	HEK293 IC_50_ (μM) ± SD	Selectivity Index
1	8.94 ± 2.18	0.70	6.43 ± 1.78	0.79	–	–
2	26.52 ± 3.85	0.23	17.90 ± 2.46	0.28	–	–
3	28.25 ± 5.46	0.22	8.19 ± 2.18	0.62	–	–
4	14.17 ± 2.75	0.44	23.25 ± 4.29	0.22	–	–
5	16.32 ± 4.24	0.38	6.51 ± 1.07	0.78	–	–
6	33.98 ± 4.98	0.18	16.13 ± 4.85	0.31	–	–
7	5.13 ± 0.94	1.21	4.60 ± 0.85	1.10	3.14 ± 0.57	0.64
8	>50	<0.12	>50	<0.10	–	–
9	>50	<0.12	>50	<0.10	–	–
12	>50	<0.12	>50	<0.10	–	–
19	3.16 ± 1.12	1.97	0.77 ± 0.21	6.56	7.33 ± 1.75	3.84
24	12.08 ± 1.99	0.51	16.43 ± 4.22	0.31	–	–
25	26.12 ± 4.55	0.24	27.85 ± 5.28	0.18	–	–
26	28.95 ± 5.86	0.21	30.85 ± 4.18	0.16	–	–
SU	6.22 ± 1.84	1.00	5.05 ± 1.34	1.00	12.19 ± 2.83	2.14

**Table 2:** Activities of compounds 19 and 27 on isolated kinases and on cell viability.

Kinase profiling					
Kinase	19		27		Potency improvement
	K_d_ (nM)	K_dRTK_/K_dKIT_	K_d_ (nM)	K_dRTK_/K_dKIT_	K_d_19/K_d_27
CSF1R	2900	132	1100	110	2.6
KIT	22	1	10	1	2.2
FLT3	4600	209	1300	130	3.5
PDGFRα	960	44	110	11	8.7
PDGFRβ	80	4	70	7	1.1
**Cytotoxicity**					
	**19**		**27**		**Sunitinib**
	**IC_50_ (μM) ± SD**		**IC_50_ (μM) ± SD**		**IC_50_ (μM) ± SD**
A549	3.16 ± 1.12		3.11 ± 1.09		12.19 ± 2.83
BxPC3	0.77 ± 0.21		0.81 ± 0.32		5.05 ± 1.34
2008	1.98 ± 0.75		1.82 ± 0.73		3.52 ± 1.22
MCF-7	1.23 ± 0.89		1.55 ± 0.75		2.58 ± 0.85
A431	2.85 ± 1.03		1.96 ± 0.92		3.89 ± 1.15
LoVo	7.45 ± 1.53		7.76 ± 1.28		8.18 ± 2.67
A375	6.41 ± 2.02		5.56 ± 1.39		8.25 ± 2.88
