# Supplementary material for: Targeting kinases with anilinopyrimidines: discovery of N-phenyl-N’-[4-(pyrimidin-4-ylamino)phenyl]urea derivatives as selective inhibitors of class III receptor tyrosine kinase subfamily
Source: Sci Rep. 2015 Nov 16;5:16750. doi: 10.1038/srep16750 (PMC4645160; doi:10.1038/srep16750)
Supplement: Supplementary Information [file srep16750-s1.pdf]

Targeting kinases with anilinopyrimidines: discovery of *N*-phenyl-*N'*-[4-(pyrimidin-4-ylamino)phenyl]urea derivatives as selective inhibitors of class III receptor tyrosine kinase subfamily

Valentina Gandin, Alessandro Ferrarese, Martina Dalla Via, Cristina Marzano, Adriana Chilin and Giovanni Marzaro*

Department of Pharmaceutical and Pharmacological Sciences, University of Padova, via Marzolo , I-35131, Padova (Italy)

*Phone: +39 049 8275024; Fax: +39 049 8275366; E-mail: giovanni.marzaro@unipd.it.

Supplementary Information

This file includes:

1. **Supplementary Material and Methods (page S2 to S27)**
2. **Supplementary Data (page S28 to S40)**
3. **Supplementary References (page S41)**

Supplementary Material and Methods

Part I: Synthesis of the potential TKIs

Table S1. Structures of synthesized compounds.

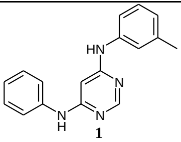 **1**	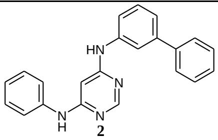 **2**	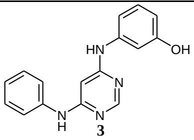 **3**	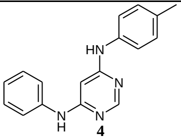 **4**
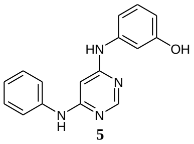 **5**	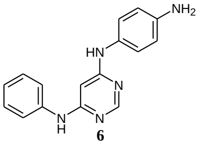 **6**	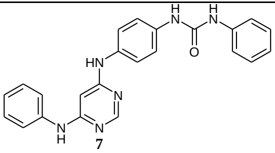 **7**	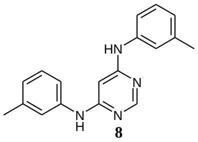 **8**
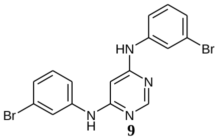 **9**	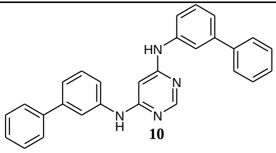 **10**	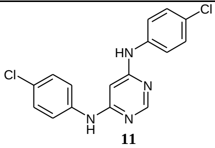 **11**	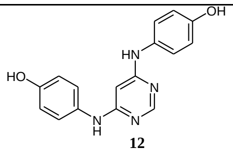 **12**
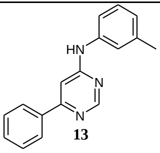 **13**	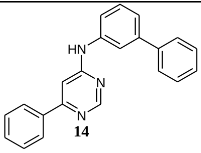 **14**	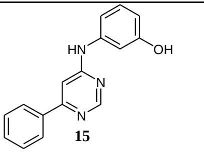 **15**	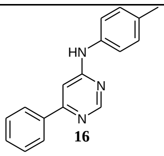 **16**
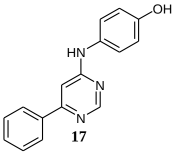 **17**	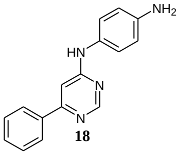 **18**	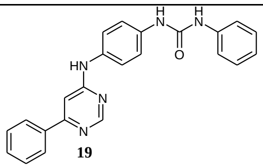 **19**	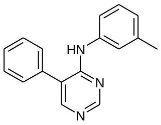 **20**
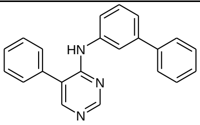 **21**	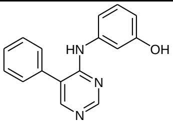 **22**	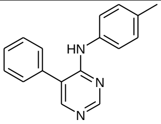 **23**	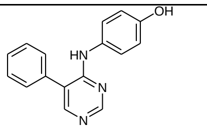 **24**
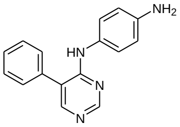 **25**		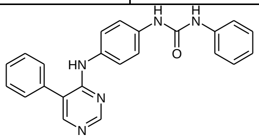 **26**	

Table S2. Recent literature on closely related compounds

General structure of known compounds	Type	Reference
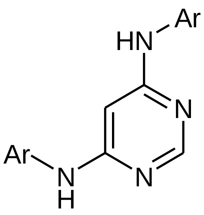	Article	1
	Article	2
	Article	3
	Article	4
	Patent	US5880130
	Patent	US6632820
	Patent	WO2006/61415 A1
	Patent	WO2007/56151
	Patent	WO2009/51822
	Patent	WO2011/88027
	Patent	WO2014/183300
	Patent	WO2015/6492
	Patent	US2015/45370
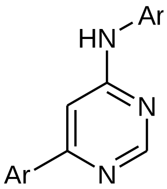	Article	2
	Article	4
	Article	5
	Article	6
	Article	7
	Article	8
	Patent	US2004/204386
	Patent	WO2005/26129
	Patent	WO2005/33086
	Patent	WO2005/70900
	Patent	WO2007/56151
	Patent	WO2008/129080
	Patent	WO2008/33834
	Patent	US2011/21524
	Patent	US2014/57911
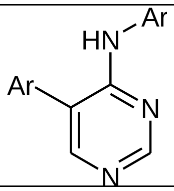	Article	9
	Patent	WO2011/99764

Experimental chemistry methods

All commercial chemicals and solvents used were analytical grade and were used without further purification. Analytical thin layer chromatography (tlc) was performed on pre-coated silica gel plates (Merck 60-F-254, 0.25 mm). Flash chromatography was performed on a Biotage Isolera One using SNAP KP-Sil cartridges, eluting with the solvent specified for individual compounds. Melting points were determined on a Gallenkamp MFB-595-010M melting point apparatus and are uncorrected. The ^{1}H-NMR spectra were recorded on a Bruker 300-AMX spectrometer with TMS as an internal standard. Coupling constants are given in Hz, and the relative area peaks were in agreement with all assignments. Elemental analyses were performed on a Perkin-Elmer 2400 analyzer. Mass spectra were performed on an Applied Biosystem Mariner System 5220 with direct injection of the sample. Microwave assisted reactions were performed on a CEM Discover® monomode reactor with the temperature monitored by a built-in infrared sensor and automatic control of power; all reactions were performed in closed devices with pressure control. Purity for all the tested compounds was determined by elemental analyses and found to be equal to or greater than 95%.

Synthesis of 6-anilino-4-phenylaminopyrimidines 1-7.

Scheme S1. Synthesis of compounds **1-7** and **8-12**[a]

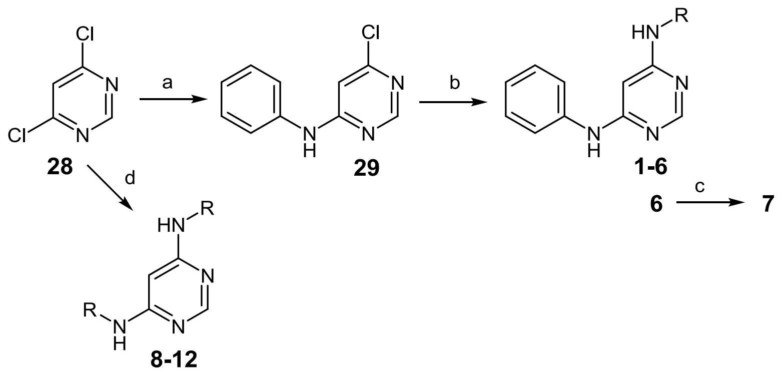

[a]Reagents and conditions: (a) Aniline, TEA, *i*-PrOH, MW, 150 °C, 30 min; (b) Aniline derivative, *i*-PrOH, MW, 150 °C, 20 min; (c) Phenyl isocyanate, CH_2Cl_2, RT, 16 h.; (d) Aniline derivative, *i*-PrOH, reflux, 24 h. See Table S1 for R specification.

4-Chloro-6-anilinopyrimidine (29). A mixture of **28** (1.3 g, 9.0 mmol), aniline (1.0 mL, 9.9 mmol) and TEA (1.4 mL, 18.0 mmol) in *i*-PrOH (3 mL) was microwave irradiated at 150 °C (power set point 100 W; ramp time 1 min; hold time 30 min). After cooling, the mixture was evaporated under reduced pressure. The residue was diluted with sat. $NaHCO_3$ solution (20 mL) and the resulting precipitate was collected by filtration to give **29** (1.6 g, 84%); mp: 156°C. ^{1}H NMR (300 MHz, DMSO-d_6): δ 9.88 (broad s, 1H, NH), 8.47 (s, 1H, 2-H), 7.61 (d, J = 8.3 Hz, 2H, 1'-H and 6'-H), 7.37 (dd, J = 7.8, 7.4 Hz, 2H, 2'-H and 4'-H), 7.08 (t, J = 7.4 Hz, 1H, 3'-H), 6.79 (s, 1H, 5-H). Anal. calcd. for $C_{10}H_8ClN_3$: C, 58.41; H, 3.92; Cl, 17.24; N, 20.43; found: C, 58.44; H, 3.94; Cl, 17.26; N, 20.44.

General procedure for 6-anilino-4-phenylaminopyrimidines 1-6.

A mixture of **29** (0.2 g, 1.0 mmol) and aniline derivative (1.0 mmol) in *i*-PrOH (3 mL) was microwave irradiated at 150 °C (power set point 200 W; ramp time 1 min; hold time 20 min).

After cooling, the mixture was poured into sat. $NaHCO_3$ (20 mL) and the obtained precipitate was collected by filtration. The solid was crystallized to give **1-6.**

4-(3''-Methyl)aniline-6-phenylaminopyrimidine (1). From *m*-toluidine, yield 20% ($CHCl_3$); mp: 254 °C. ^{1}H NMR (300 MHz, DMSO-*d*$_6$): δ 9.13 (s, 1H, NH), 9.04 (s, 1H, NH), 8.26 (s, 1H, 2-H), 7.57-7.52 (m, 2H, Ar-H), 7.37-7.26 (m, 4H, Ar-H), 7.17 (t, *J* = 7.6 Hz, 1H, 5''-H), 6.96-6.94 (m, 1H, Ar-H), 6.79 (d, *J* = 7.6 Hz, 1H, 4''-H), 6.17 (s, 1H, 5-H), 2.28 (s, 3H, CH_3). ^{13}C NMR (75 MHz, DMSO-*d*$_6$): δ 160.49, 160.43, 157.59, 140.36, 140.23, 137.78, 128.60, 128.47, 122.56, 121.69, 120.38, 119.78, 117.07, 86.01, 21.15. Anal. calcd. for $C_{17}H_{16}N_4$: C, 73.89; H, 5.84; N, 20.27; found: C, 73.86; H, 5.85; N, 20.29. HRMS (ESI-TOF) for $C_{17}H_{17}N_4$ $[M + H]^+$: calcd, 277.1448; found, 277.1510.

4-(3''-Biphenyl)amino-6-phenylaminopyrimidine (2). From 3-aminobiphenyl, yield 76% (EtOAc); mp: 267 °C. ^{1}H NMR (300 MHz, DMSO-*d*$_6$): δ 9.25 (s, 1H, NH), 9.17 (s, 1H, NH), 8.29 (s, 1H, 2-H), 7.84-7.81 (m, 1H, 2''-H), 766-7.61 (m, 2H, Ar-H), 7.59-7.53 (m, 3H, Ar-H), 7.51-7.45 (m, 2H, Ar-H), 7.42-7.35 (m, 2H, Ar-H), 7.32-7.23 (m, 3H, Ar-H), 7.00-6.94 (m, 1H, Ar-H), 6.23 (s, 1H, 5-H). ^{13}C NMR (75 MHz, DMSO-*d*$_6$): δ 160.54, 160.48, 157.68, 140.96, 140.75, 140.34, 140.30, 129.23, 128.83, 128.62, 127.36, 126.57, 121.75, 120.16, 119.85, 118.79, 117.98, 86.25. Anal. calcd. for $C_{22}H_{18}N_4$: C, 78.08; H, 5.36; N, 16.56; found: C, 78.11; H, 5.33; N, 16.70. HRMS (ESI-TOF) for $C_{22}H_{19}N_4$ $[M + H]^+$: calcd, 339.1604; found, 339.1589.

4-(3''-Hydroxy)anilino-6-phenylaminopyrimidine (3). From 3-aminophenol, yield 24% (EtOAc/*n*-hexane); mp: 253 °C. ^{1}H NMR (300 MHz, DMSO-*d*$_6$): δ 9.31 (broad s, 1H, OH), 9.12 (s, 1H, NH), 9.01 (s, 1H, NH), 8.25 (s, 1H, 2-H), 7.57-7.52 (m, 2H, 2'-H and 6'-H), 7.32-7.26 (m, 2H, 3'-H and 5'-H), 7.10-7.03 (m, 2H, 2''-H and 5''-H), 6.99-6.94 (m, 1H, 4'-H), 6.93-6.88 (m, 1H, 4''-H or 6''-H), 6.40-6.36 (m, 1H, 4''-H or 6''-H), 6.18 (s, 1H, 5-H). ^{13}C NMR (75 MHz, DMSO-*d*$_6$): δ 160.50, 160.44, 157.62, 157.57, 141.38, 140.38, 129.23, 128.60, 121.66,

119.75, 110.65, 109.02, 106.93, 86.16. Anal. calcd. for $C_{16}H_{14}N_4O$: C, 69.05; H, 5.07; N, 20.13; found: C, 69.03; H, 5.10; N, 20.15. HRMS (ESI-TOF) for $C_{16}H_{15}N_4O$ $[M + H]^+$: calcd, 279.1240; found, 279.1224.

4-(4''-Methyl)anilino-6-phenylaminopyrimidine (4). From *p*-toluidine, yield 64% (EtOAc); mp: 265 °C. ^1H NMR (300 MHz, DMSO-d_6): δ 9.10 (s, 1H, NH), 9.02 (s, 1H, NH), 8.24 (s, 1H, 2-H), 7.57-7.51 (m, 2H, 2'-H and 6'-H), 7.40 (d, J = 8.4 Hz, 2H, 2''-H and 6''-H), 7.32-7.24 (m, 2H, 3'-H and 5'-H), 7.10 (d, J = 8.4 Hz, 2H, 3''-H and 5''-H), 6.96 (t, J = 7.4 Hz, 1H, 4'-H), 6.12 (s, 1H, 5-H), 2.26 (s, 3H, CH_3). ^{13}C NMR (75 MHz, DMSO-d_6): δ 160.61, 160.41, 157.58, 140.42, 137.66, 130.80, 129.05, 128.57, 121.58, 120.19, 119.67, 85.66, 20.27. Anal. calcd. for $C_{17}H_{16}N_4$: C, 73.89; H, 5.84; N, 20.27; found: C, 73.90; H, 5.82; N, 20.28. HRMS (ESI-TOF) for $C_{17}H_{17}N_4$ $[M + H]^+$: calcd, 277.1448; found, 277.1401.

4-(4''-Hydroxy)anilino-6-phenylaminopyrimidine (5). From 4-aminophenol, yield 78% (MeOH/DCM); mp: 286 °C. ^1H NMR (300 MHz, DMSO-d_6): δ 9.02 (s, 1H, NH), 8.71 (s, 1H, NH), 8.17 (s, 1H, 2-H), 7.54 (d, J = 8.0 Hz, 2H, 2''-H and 6''-H), 7.30-7.16 (m, 4H, 2'-H, 3'H, 5'-H and 6'-H), 6.93 (t, J = 7.4 Hz, 1H, 4'-H), 6.71 (d, J = 8.0 Hz, 2H, 3''-H and 5''-H), 5.97 (s, 1H, 5-H). ^{13}C NMR (75 MHz, DMSO-d_6): δ 161.26, 160.41, 157.62, 153.37, 140.59, 131.16, 128.54, 123.29, 121.40, 119.51, 115.31, 84.65. Anal. calcd. for $C_{16}H_{14}N_4O$: C, 69.05; H, 5.07; N, 20.13; found: C, 69.08; H, 5.09; N, 20.11. HRMS (ESI-TOF) for $C_{16}H_{15}N_4O$ $[M + H]^+$: calcd, 279.1240; found, 279.1189.

4-(4''-Amino)anilino-6-phenylaminopyrimidine (6). From 4-aminoacetanilide, the acetylamino intermediate was obtained, which was suspended in 3M HCl (5 ml) and heated at reflux for 3 h. The mixture was made alkaline with 3M NaOH, then the obtained precipitate was collected by filtration to give **6**, yield 72%; mp: 253 °C. ^1H NMR (300 MHz, DMSO-d_6): δ 8.97 (s, 1H, NH), 8.53 (s, 1H, NH), 8.13 (s, 1H, 2-H), 7.56-7.51 (m, 2H, 2'-H and 6'-H), 7.27-

7.22 (m, 2H, 3'-H and 5'-H), 7.02 (d, J = 8.6 Hz, 2H, 2''-H and 6''-H), 6.94-6.89 (m, 1H, 6'-H), 6.55 (d, J = 8.6 Hz, 2H, 3''-H and 5''-H), 5.90 (s, 1H, 5-H), 4.92 (s, 2H, NH_2). ^{13}C NMR (75 MHz, DMSO-d_6): δ 161.66, 160.34, 157.56, 145.05, 140.66, 128.47, 128.19, 123.93, 121.21, 119.33, 114.11, 84.11. Anal. calcd. for $C_{16}H_{15}N_5$: C, 69.29; H, 5.45; N, 25.25; found: C, 69.27; H, 5.47; N, 25.25. HRMS (ESI-TOF) for $C_{16}H_{16}N_5$ $[M + H]^+$: calcd, 278.1400; found, 278.1406.

***N*-phenyl-*N*'-[4'-(6''-phenylaminopyrimidin-4''-ylamino)phenyl]urea (7).** A solution of **6** (0.17 g, 0.6 mmol) in dichloromethane (5 mL) was added dropwise at 0 °C to a solution of phenyl isocyanate (71.5 mg, 0.6 mmol) in dichloromethane (2 mL) and the mixture was stirred at room temperature for 16 h. After cooling, the obtained precipitate was collected by filtration and purified by flash chromatography (eluting with $CHCl_3$/MeOH, 9/1) to give **7**, yield 34%; mp: >300 °C. ^{1}H NMR (300 MHz, DMSO-d_6): δ 9.08 (s, 1H, NH), 8.98 (s, 1H, NH), 8.69 (broad s, 1H, NH), 8.66 (broad s, 1H, NH), 8.23 (s, 1H, 2''-H), 7.57-7.51 (m, 2 H, Ar-H), 7.47-7.36 (m, 6 H, Ar-H), 7.32-7.23 (m, 4 H, Ar-H), 6.98-6.92 (m, 2 H, Ar-H), 8.10 (s, 1 H, 5''-H). ^{13}C NMR (75 MHz, DMSO-d_6): δ 160.71, 160.39, 157.60, 152.51, 140.45, 139.75, 134.43, 134.35, 128.63, 128.56, 121.53, 121.13, 119.61, 118.85, 117.99, 85.37. Anal. calcd. for $C_{23}H_{20}N_5O$: C, 69.68; H, 5.08; N, 21.20; found: C, 69.70; H, 5.04; N, 21.19. HRMS (ESI-TOF) for $C_{23}H_{21}N_6O$ $[M + H]^+$: calcd, 397.1771; found, 397.1721.

Synthesis of 4,6-dianilinopyrimidines 8-12.

General procedure for 4,6-dianilinopyrimidines 8-12. A mixture of **28** (0.2 g, 1.0 mmol) and aniline derivative (2.0 mmol) in *i*-PrOH (5 mL) was heated at reflux for 24 h. After cooling, the mixture was poured into water (30 mL) and the obtained precipitate was collected by filtration. The solid was crystallized from *i*-PrOH to give **8-12**.

4,6-Di-[(3'-methyl)anilino]pyrimidine (8). From *m*-toluidine, yield 51%; mp: dec. at 190 °C. ^{1}H NMR (300 MHz, DMSO-d_6): δ 9.05 (s, 2H, NH), 8.25 (s, 1H, 2-H), 7.38-7.30 (m, 4H, 2'-H and 4'-H or 6'-H), 7.17 (t, *J* = 7.3 Hz, 2H, 5'-H), 6.79 (d, *J* = 7.3 Hz, 2H, 4'-H or 6'-H), 6.16 (s, 1H, 5-H), 2.28 (s, 6H, CH_3). ^{13}C NMR (75 MHz, DMSO-d_6): δ 160.46, 157.56, 140.23, 137.75, 128.44, 122.53, 120.37, 117.06, 85.89, 21.12. Anal. calcd. for $C_{18}H_{18}N_4$: C, 74.46; H, 6.25; N, 19.30; found: C, 74.47; H, 6.29; N, 19.29. HRMS (ESI-TOF) for $C_{18}H_{19}N_4$ $[M + H]^+$: calcd, 291.1604; found, 291.1567.

4,6-Di-[(3'-bromo)anilino]pyrimidine (9). From 3-bromoaniline, yield 60%; mp: 243 °C. ^{1}H NMR (300 MHz, DMSO-d_6): δ 9.42 (s, 2H, NH), 8.38 (s, 1H, 2-H), 8.00 (t, *J* = 2.0 Hz, 2H, 2'-H), 7.50 (dt, *J* = 8.1 Hz , 2.0 Hz, 2H, 4'-H or 6'-H), 7.25 (t, J = 8.1 Hz, 2H, 4'-H), 7.13 (dt, J = 8.1 Hz, 2.0 Hz, 2H, 4'-H or 6'-H), 6.18 (s, 1H, 5-H). ^{13}C NMR (75 MHz, DMSO-d_6): δ 160.05, 157.53, 142.10, 130.46, 123.90, 121.49, 121.44, 117.98, 87.91. Anal. calcd. for $C_{16}H_{12}Br_2N_4$: C, 45.74; H, 2.88; Br, 38.04; N, 13.34; found: C, 45.77; H, 2.89; Br, 38.01; N, 13.36. HRMS (ESI-TOF) for $C_{16}H_{13}Br_2N_4$ $[M + H]^+$: calcd, 418.9507; found, 418.9455; $[M + 2 + H]^+$: calcd, 420.9487; found, 420.9396; $[M + 4 + H]^+$: calcd, 422.9466; found, 422.9368.

4,6-Di-[(3'-biphenyl)amino]pyrimidine (10). From 3-aminobiphenyl, yield 6%; mp: 273 °C. ^{1}H NMR (300 MHz, DMSO-d_6): δ 9.28 (s, 2H, NH), 8.31 (s, 1H, 2-H), 7.82 (t, *J* = 3.4 Hz, 2H, Ar-H), 7.68-7.22 (m, 16H, Ar-H), 6.27 (s, 1H, 5-H). ^{13}C NMR (75 MHz, DMSO-d_6): δ 160.49, 157.71, 140.90, 140.76, 140.28, 129.24, 128.85, 127.39, 126.58, 120.22, 118.82, 118.03,

87.84. Anal. calcd. for $C_{28}H_{22}N_4$: C, 81.13; H, 5.35; N, 13.52; found: C, 81.14; H, 5.37; N, 13.50. HRMS (ESI-TOF) for $C_{28}H_{23}N_4$ $[M + H]^+$: calcd, 415.1917; found, 415.1890.

4,6-Di-[(4'-chloro)anilino]pyrimidine (11). From 4-chloroaniline, yield 57%; mp: 296 °C. ^{1}H NMR (300 MHz, DMSO-d_6): δ 9.33 (s, 2H, NH), 8.31 (s, 1H, 2-H), 7.62 (d, *J* = 8.9 Hz, 4H, 3'-H and 5'-H), 7.33 (d, *J* = 8.9 Hz, 4H, 2'-H and 6'-H), 6.15 (s, 1H, 5-H). ^{13}C NMR (75 MHz, DMSO-d_6): δ 160.16, 157.48, 139.39, 128.40, 125.03, 120.94, 87.19. Anal. calcd. for $C_{16}H_{12}Cl_2N_4$: C, 58.02; H, 3.65; Cl, 21.41; N, 16.92; found: C, 58.05; H, 3.67 Cl, 21.40; N, 16.89. HRMS (ESI-TOF) for $C_{16}H_{13}Cl_2N_4$ $[M + H]^+$: calcd, 331.0517; found, 331.0484; $[M + 2 + H]^+$: calcd, 333.0488; found, 333.0457.

4,6-Di-[(4'-hydroxy)anilino]pyrimidine (12). From 4-aminophenol, yield 14%; mp: dec. at 255 °C. ^{1}H NMR (300 MHz, DMSO-d_6): δ 9.22 (s, 2H, NH), 8.87 (broad s, 2H, OH), 8.12 (s, 1H, 2-H), 7.19 (d, *J* = 7.0 Hz, 4H, 3'-H and 5'-H), 6.71 (d, *J* = 7.0 Hz, 4H, 2'-H and 6'-H), 5.79 (s, 1H, 5-H). ^{13}C NMR (75 MHz, DMSO-d_6): δ 159.98, 153.60, 130.46, 123.51, 115.32, 82.55. Anal. calcd. for $C_{16}H_{14}N_4O_2$: C, 65.30; H, 4.79; N, 19.04; found: C, 65.31; H, 4.79; N, 19.01. HRMS (ESI-TOF) for $C_{16}H_{15}N_4O_2$ $[M + H]^+$: calcd, 295.1190; found, 295.1167.

Synthesis of 4-anilino-6-phenylpyrimidines 13-19.

Scheme S2. Synthesis of compounds **13-19**[a]

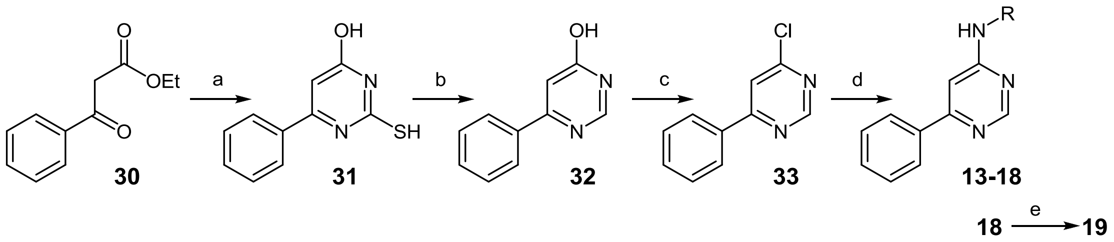

[a]Reagents and conditions: (a) Thiourea, EtONa/EtOH, reflux, 24 h; (b) Raney-Ni, NH_4OH/EtOH (3/1), reflux, 6 h; (c) $POCl_3$, TEA, reflux, 1 h; (d) Aniline derivative, *i*-PrOH, MW, 150 °C, 20 min; (e) Phenyl isocyanate, CH_2Cl_2, RT, 16 h. See Table S1 for R specification.

4-Hydroxy-6-phenyl-2-mercaptopyrimidine (31). To a solution of ethyl benzoylacetate (**30**) (8.6 mL, 50.0 mmol) in EtOH (30 mL), an EtONa/EtOH 20% solution (20 ml) was added and the mixture was stirred at room temperature for 20 min. Thiourea (4.6 g, 60.0 mmol) was then added, and the mixture was heated at reflux for 24 h. After cooling, the mixture was evaporated under reduced pressure, and the residue was diluted with water (100 mL) and acidified with conc. HCl to pH = 2. The obtained precipitate was collected by filtration and washed with water to give **31** (9.0 g, 88%); mp: 265 °C. ^{1}H NMR (300 MHz, DMSO-d_6): δ 12.52 (broad s, 1H, SH or OH), 12.49 (broad s, 1H, SH or OH), 7.73-7.67 (m, 2H, 2'-H and 6'-H), 7.59-7.45 (m, 3H, 5'-H, 3'-H and 4'-H), 6.08 (d, *J* = 0.1 Hz, 1H, 5-H). Anal. calcd. for $C_{10}H_8N_2OS$: C, 58.81; H, 3.95; N, 13.72; S, 15.70; found: C, 58.84; H, 3.96; N, 13.74; S, 15.68.

4-Hydroxy-6-phenylpyrimidine (32). To a solution of **31** (4.1 g, 20.0 mmol) in ethanolic NH_4OH (3:1, 300 mL) an aqueous suspension of Raney-Nickel (8 mL) was added and the mixture was heated at reflux for 6 h. The mixture was evaporated under reduced pressure, and the residue was diluted with water (100 mL) and acidified with conc. HCl to pH = 2. The

obtained precipitate was collected by filtration and washed with water to give **32** (2.1 g, 60%); mp: 274 °C. ^{1}H NMR (300 MHz, DMSO-d_6): δ 12.53 (broad s, 1H, OH), 8.27 (s, 1H, 2-H), 8.04-8.02 (m, 2H, 2'-H and 6'-H), 7.51-7.46 (m, 3 H, 5'-H, 3'-H and 4'-H), 6.88 (s, 1H, 5-H). Anal. calcd. for $C_{10}H_8N_2O$: C, 69.76; H, 4.68; N, 16.27; found: C, 69.75; H, 4.64; N, 16.78.

4-Chloro-6-phenylpyrimidine (33). To a mixture of **32** (2.0 g, 11.6 mmol) and phosphorus oxychloride (5.0 mL, 50.0 mmol), TEA (3.0 mL, 20.0 mmol) was added portionwise under stirring at room temperature over a period of 1 h. The mixture was heated at reflux for 1 h and then the solvent was removed under nitrogen stream. The residue was diluted with water (100 mL) and the obtained precipitate was collected by filtration to give **33** (2.0 g, 90%); mp: 156 °C. ^{1}H NMR (300 MHz, DMSO-d_6): δ 9.10 (s, 1H, 2-H), 8.33 (s, 1H, 5-H), 8.28-8.23 (m, 2H, 2'-H and 6'-H), 7.63-7.53 (m, 3H, 3'-H, 4'-H and 5'-H). Anal. calcd. for $C_{10}H_7ClN_2$: C, 63.01; H, 3.70; Cl, 18.60; N, 14.70; found: C, 62.99; H, 3.72; Cl, 18.62; N, 14.67.

General procedure for 4-anilino-6-phenylpyrimidines 13-18.

A mixture of **33** (0.2 g, 1.0 mmol) and aniline derivative (1.0 mmol) in *i*-PrOH (3 mL) was microwave irradiated at 150 °C (power set point 200 W; ramp time 1 min; hold time 20 min). After cooling, the resulting precipitate was collected by filtration to give **13-17** (as hydrochlorides) and **18**.

4-(3''-Methyl)anilino-6-phenylpyrimidine hydrochloride (13). From *m*-toluidine, yield 71%; mp: 272 °C. ^{1}H NMR (300 MHz, DMSO-d_6): δ 10.61 (broad s, 1H, NH), 8.86 (s, 1H, 2-H), 8.00-7.91 (m, 2H, 2'-H and 6'-H), 7.66-7.56 (m, 3H, 3'-H, 4'-H and 5'-H), 7.55-7.46 (m, 2H, 2''-H and 6''-H), 7.35-7.25 (m, 2H, 5-H and 5''-H), 6.99 (d, J = 7.19 Hz, 1H, 4''-H), 2.34 (s, 3H, CH_3). ^{13}C NMR (75 MHz, DMSO-d_6): δ 162.03, 156.98, 154.17, 153.92, 138.89, 137.95,

132.63, 129.89, 129.36, 127.74, 126.40, 122.67, 119.47, 103.77, 21.63. Anal. calcd. for $C_{17}H_{15}N_3$•HCl: C, 68.57; H, 5.42; Cl, 11.91; N, 14.11; found: C, 68.55; H, 5.44; Cl, 11.90; N, 14.14. HRMS (ESI-TOF) for $C_{17}H_{16}N_3$ $[M + H]^+$: calcd, 262.1339; found, 262.1324.

4-(3''-Biphenyl)amino-6-phenylpyrimidine hydrochloride (14). From 3-aminobiphenyl, yield 71%; mp: 242 °C. ^{1}H NMR (300 MHz, DMSO-*d*$_6$): δ 10.40 (broad s, 1H, NH), 8.84 (s, 1H, 2-H), 8.05-7.93 (m, 3H, Ar-H), 7.56-7.33 (m, 11H, Ar-H), 7.30 (s, 1H, 5-H). ^{13}C NMR (75 MHz, DMSO-*d*$_6$): δ 161.51, 153.52, 153.08, 140.92, 139.56, 138.05, 132.09, 130.56, 129.48, 129.29, 128.89, 127.65, 127.16, 126.59, 123.36, 120.56, 119.90, 103.42. Anal. calcd. for $C_{22}H_{17}N_3$•HCl: C, 73.43; H, 5.04; Cl, 9.85; N, 11.68; found: C, 73.41; H, 5.03; Cl, 9.85; N, 11.71. HRMS (ESI-TOF) for $C_{22}H_{18}N_3$ $[M + H]^+$: calcd, 324.1495; found, 324.1484.

4-(3''-Hydroxy)anilino-6-phenylpyrimidine hydrochloride (15). From 3-aminophenol, yield 77%; mp: 273 °C. ^{1}H NMR (300 MHz, DMSO-*d*$_6$): δ 10.53 (broad s, 1H, NH), 8.85 (s, 1H, 2-H), 8.01-7.87 (m, 2H, 2'-H and 6'-H), 7.66-7.55 (m, 3H, 3'-H, 4'-H and 5'-H), 7.28 (s, 1H, 5-H), 6.96-7.06 (m, 3H, 2''-H, 5''-H and 6''-H), 6.61-6.53 (m, 1H, 4''-H). ^{13}C NMR (75 MHz, DMSO-*d*$_6$): δ 161.41, 157.84, 153.27, 152.85, 138.24, 132.17, 130.47, 129.65, 129.35, 127.25, 112.51, 112.42, 108.82, 103.28. Anal. calcd. for $C_{16}H_{13}N_3O$•HCl: C, 64.11; H, 4.71; Cl, 11.83; N, 14.02; found: C, 64.10; H, 4.73; Cl, 11.85; N, 14.00. HRMS (ESI-TOF) for $C_{16}H_{14}N_3O$ $[M + H]^+$: calcd, 264.1131; found, 264.1086.

4-(4''-Methyl)anilino-6-phenylpyrimidine hydrochloride (16). From *p*-toluidine, yield 84%; mp: 276 °C. ^{1}H NMR (300 MHz, DMSO-*d*$_6$): δ 10.32 (broad s,1 H, NH), 8.80 (s, 1H, 2-H), 7.99-7.89 (m, 2H, 2'-H and 6'-H), 7.63-7.52 (m, 5H, Ar-H), 7.26-7.16 (m, 3H, Ar-H and 5-H), 2.30 (s, 3H, CH_3). ^{13}C NMR (75 MHz, DMSO-*d*$_6$): δ 161.34, 153.59, 153.29, 134.83, 134.51, 132.06, 130.86, 129.43, 129.33, 127.19, 121.77, 102.96, 20.50. Anal. calcd. for

$C_{17}H_{15}N_3$•HCl: C, 68.57; H, 5.42; Cl, 11.91; N, 14.11; found: C, 68.54; H, 5.40; Cl, 11.94; N, 14.10. HRMS (ESI-TOF) for $C_{17}H_{16}N_3$ $[M + H]^+$: calcd, 262.1339; found, 262.1304.

4-(4''-Hydroxy)anilino-6-phenylpyrimidine hydrochloride (17). From 4-aminophenol, yield 90%; mp: 270 °C. ^{1}H NMR (300 MHz, DMSO-*d*$_6$): δ 10.62 (broad s, 1H, OH), 9.53 (broad s, 1H, NH), 8.79 (s, 1 H, 2-H), 7.97-7.85 (m, 2H, 2'-H and 6'-H), 7.66-7.56 (m, 3H, 3'-H, 4'-H and 5'-H), 7.41 (d, *J* = 8.7 Hz, 2H, 2''-H and 6''-H), 7.12 (s, 1H, 5-H), 6.82 (d, *J* = 8.7 Hz, 2 H, 3''-H and 5''-H). ^{13}C NMR (75 MHz, DMSO-*d*$_6$): δ 161.10, 155.42, 153.14, 152.43, 132.10, 130.49, 129.33, 129.04, 128.31, 127.19, 123.89, 115.53. Anal. calcd. for $C_{16}H_{13}N_3O$•HCl: C, 64.11; H, 4.71; Cl, 11.83; N, 14.02; found: C, 64.12; H, 4.70; Cl, 11.84; N, 13.99. HRMS (ESI-TOF) for $C_{16}H_{14}N_3O$ $[M + H]^+$: calcd, 264.1131; found, 264.1048.

4-(4''-Amino)anilino-6-phenylpyrimidine (18). From 4-aminoacetanilide, the acetylamino intermediate was obtained, which was suspended in 3M HCl (5 ml) and heated at reflux for 3 h. The mixture was made alkaline with 3M NaOH, then the obtained precipitate was collected by filtration to give **18**, yield 95%; mp: 210 °C. ^{1}H NMR (300 MHz, DMSO-*d*$_6$): δ 9.18 (broad s, 1H, NH), 8.56 (s, 1H, 2-H), 8.02-7.89 (m, 2H, 2'-H and 6'-H), 7.55-7.45 (m, 3H, 3'-H, 4'-H and 5'-H), 7.21 (d, *J* = 8.4, 2H, 2''-H and 6''-H), 7.00 (s, 1H, 5-H), 6.57 (d, *J* = 8.4, 2H, 3''-H and 5''-H), 4.95 (broad s, 2H, NH_2). ^{13}C NMR (75 MHz, DMSO-*d*$_6$): δ 161.50, 160.67, 158.35, 145.12, 137.15, 130.00, 128.73, 126.22, 123.01, 114.04, 83.28. Anal. calcd. for $C_{16}H_{14}N_4$: C, 73.26; H, 5.38; N, 21.36; found: C, 73.27; H, 5.40; N, 21.35. HRMS (ESI-TOF) for $C_{16}H_{15}N_4$ $[M + H]^+$: calcd, 263.1291; found, 263.1301.

***N*-phenyl-*N*'-[4'-(6''-phenylpyrimidin-4''-ylamino)phenyl]urea (19).** A solution of **18** (0.16 g, 0.6 mmol) in dichloromethane (5 mL) was added dropwise at 0 °C to a solution of phenyl isocyanate (71.5 mg, 0.6 mmol) in dichloromethane (2 mL) and the mixture was stirred at

room temperature for 16 h. After cooling, the obtained precipitate was collected by filtration and crystallized from MeOH to give **19**, yield 29%; mp: 295 °C. ^{1}H NMR (300 MHz, DMSO-d_6): δ 10.96 (broad s, 1H, NH), 9.10 (s, 1H, NH), 9.00 (s, 1H, NH), 8.87 (s, 1H, 2''-H), 7.95-7.89 (m, 2H, Ar-H), 7.68-7.55 (m, 5H, Ar-H), 7.52 (d, *J* = 9.0 Hz, 2H, 2'-H and 6'-H or 3'-H and 5'-H), 7.46 (d, *J* = 9.0 Hz, 2H, 2'-H and 6'-H or 3'-H and 5'-H), 7.31-7.25 (m, 2H, ArH), 7.23 (s, 1H, 5''-H), 6.96 (t, *J* = 7.2 Hz, 1H, Ar-H). ^{13}C NMR (75 MHz, DMSO-d_6): δ 161.16, 153.67, 153.52, 152.54, 139.67, 137.34, 131.95, 131.07, 129.28, 128.64, 127.12, 122.73, 121.61, 118.36, 117.91. Anal. calcd. for $C_{23}H_{19}N_5O$: C, 72.42; H, 5.02; N, 18.36; found: C, 72.43; H, 4.99; N, 18.34. HRMS (ESI-TOF) for $C_{23}H_{20}N_5O$ $[M + H]^+$: calcd, 382.1662; found, 382.1684.

Synthesis of 4-anilino-5-phenylpyrimidines 20-26.

Scheme S3. Synthesis of compounds **20-26**[a]

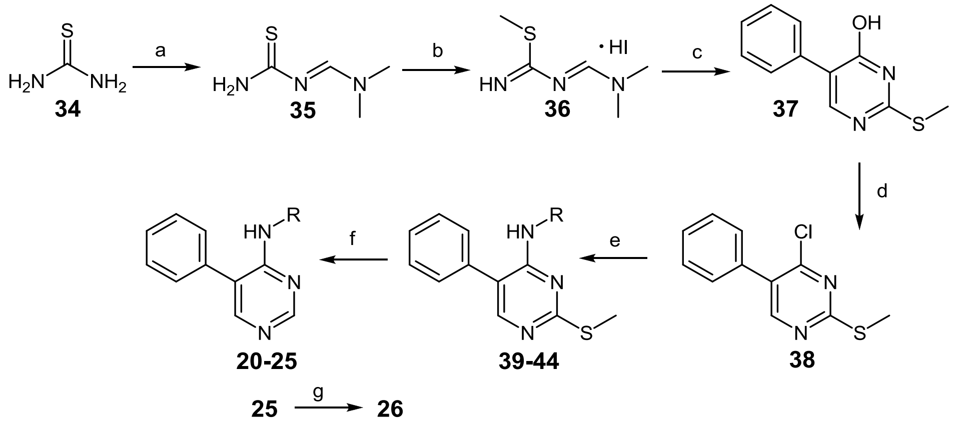

[a]Reagents and conditions: (a) *N,N*-dimethylformamide dimethyl acetal, MeOH, reflux, 4 h; (b) CH_3I, THF, RT, 6 h; (c) Phenylacetyl chloride, TEA, CH_2Cl_2, RT, 3 h; (d) $POCl_3$, TEA, reflux, 1 h; (e) Aniline derivative, *i*-PrOH, MW, 150 °C, 20 min; (f) Raney-Ni, EtOH, reflux, 2 h; (g) Phenyl isocyanate, CH_2Cl_2, RT, 16 h. See Table S1 for R specification.

***N,N*-Dimethylaminomethylene thiourea (35).** A solution of thiourea (**34**) (3.8 g, 50.0 mmol) and *N,N*-dimethylformamide dimethylacetal (7.2 g, 60.0 mmol) in MeOH (100 mL) was heated at reflux for 4 h. After cooling, the obtained precipitate was collected by filtration to give **35** (4.6 g, 70%); mp: 163 °C. ^{1}H NMR (300 MHz, DMSO-d_6): δ 8.20 (s, 1H, NH), 7.93 (s, 1H, NH), 3.33 (s, 1H, CH), 3.13 (s, 3H, CH_3), 2.98 (s, 3H, CH_3). Anal. calcd. for $C_4H_9N_3S$: C, 36.62; H, 6.91; N, 32.03; S, 24.44; found: C, 36.64; H, 6.89; N, 32.00; S, 24.41.

***N,N*-Dimethylaminomethylene-2-methylisothiourea hydroiodide (36).** A mixture of **35** (4.6 g, 35.0 mmol) and iodomethane (2.3 mL, 37.0 mmol) in THF (62 mL) was stirred at room temperature for 6 h. The mixture was evaporated under reduced pressure to give **36** (7.2 g, 96%); mp: 156 °C. ^{1}H NMR (300 MHz, DMSO-d_6): δ 9.57 (s, 2H, NH and HI); 8.39 (s, 1H, CH), 3.27 (s, 3H, N-CH_3), 3.13 (s, 3H, N-CH_3), 2.56 (s, 3H, S-CH_3). Anal. calcd. for

$C_5H_{11}N_3S$•HI: C, 21.99; H, 4.43; I, 46.46; N, 15.38; S, 11.74; found: C, 22.01; H, 4.45; I, 46.44; N, 15.36; S, 11.73.

4-Hydroxy-2-methylthio-5-phenylpyrimidine (37). To a suspension of **36** (6.0 g, 22.0 mmol) in CH_2Cl_2 (50 mL) cooled at 0 °C, TEA (12.5 mL, 88.5 mmol) and phenylacetyl chloride (3.5 mL, 26.5 mmol) were added. The mixture was slowly brought at room temperature and stirred for 3 h. The mixture was extracted with 3M NaOH (3 x 50 ml) and the aqueous phase was acidified to pH = 4 with 5M HCl. The obtained precipitate was collected by filtration and washed with water to give **37** (2.6 g, 54%); mp: 250 °C. ^{1}H NMR (300 MHz, DMSO-*d$_6$*): δ 13.02 (s, 1H, OH), 8.07 (s, 1H, 6-H), 7.65 (m, 2H, 2'-H and 6'-H), 7.36 (m, 3H, 3'-H, 4'-H and 5'-H), 2.53 (s, 3H, CH_3). Anal. calcd. for $C_{11}H_{10}N_2OS$: C, 60.53; H, 4.62; N, 12.83; S, 14.69; found: C, 60.55; H, 4.61; N, 12.85; S, 14.68.

4-Chloro-2-methylthio-5-phenylpyrimidine (38). Compound **38** was obtained from **37** (2.5 g, 11.5 mmol) with the same procedure as for **33**. Yield 87%; mp: 94 °C. ^{1}H NMR (300 MHz, DMSO-*d$_6$*): δ 8.67 (s, 1H, 6-H), 7.53 (m, 5H, Ar-H), 2.58 (s, 1H, S-CH_3). Anal. calcd. for $C_{11}H_9ClN_2S$: C, 55.81; H, 3.83; Cl, 14.98; N, 11.83; S, 13.54; found: C, 55.82; H, 3.83; Cl, 14.97; N, 11.86; S, 13.55.

General procedure for 4-anilino-2-methylthio-5-phenylpyrimidines 39-44. A mixture of **38** (0.2 g, 1.0 mmol) and aniline derivative (1.0 mmol) in *i*-PrOH (3 mL) was microwave irradiated at 150 °C (power set point 150 W; ramp time 1 min; hold time 20 min). After cooling, the mixture was poured into sat. $NaHCO_3$ (20 mL) and extracted with EtOAc (3 x 20 mL). The combined organics were evaporated under reduced pressure to give **39-44**.

4-(3''-Methyl)anilino-2-methylthio-5-phenylpyrimidine (39). From *m*-toluidine, yield 94%; mp: 114 °C. ^{1}H NMR (300 MHz, DMSO-d_6): δ 8.98 (s, 1H, NH), 8.11 (s, 1H, 6-H), 8.10 (s, 1H, 2'-H), 7.86 (d, J = 8.0 Hz, 1H, 4'-H), 7.56-7.44 (m, 6H, 5'-H and Ar-H), 7.39 (d, J = 8.0 Hz, 1H, 6'-H), 2.46 (s, 3H, S-CH_3), 2.25 (s, 3H, CH_3). Anal. calcd. for $C_{18}H_{17}N_3S$: C, 70.33; H, 5.57; N, 13.67; S, 10.43; found: C, 70.35; H, 5.54; N, 13.69; S, 10.45.

4-(3''-Biphenyl)amino-2-methylthio-5-phenylpyrimidine (40). From 3-aminobiphenyl, yield 95%; mp: 102 °C. ^{1}H NMR (300 MHz, DMSO-d_6): δ 8.29 (s, 1H, NH), 8.10 (s, 1H, 6-H), 7.88 (s, 1H, 2'-H), 7.56-7.44 (m, 13H, Ar-H), 2.44 (s, 3H, CH_3). Anal. calcd. for $C_{23}H_{19}N_3S$: C, 74.77; H, 5.18; N, 11.37; S, 8.68; found: C, 74.75; H, 5.21; N, 11.37; S, 8.70.

4-(3''-Hydroxy)anilino-2-methylthio-5-phenylpyrimidine (41). From 3-aminophenol, yield 95%; mp: 159 °C. ^{1}H NMR (300 MHz, $CDCl_3$-d): δ 8.04 (s, 1H, 6-H), 7.57-7.39 (m, 5H, Ar-H), 7.37-7.33 (m, 1H, 2''-H), 7.14 (t, J = 8.0 Hz, 1H, 5''-H), 6.96-6.90 (m, 1H, 6''-H), 6.79 (broad s, 1H, NH or OH), 6.58-6.52 (m, 1H, 4''-H), 5.15 (broad s, 1 H, NH or OH), 2.59 (s, 3H, CH_3). Anal. calcd. for $C_{17}H_{15}N_3OS$: C, 66.00; H, 4.89; N, 13.58; S, 10.36; found: C, 66.02; H, 4.89; N, 13.57; S, 10.39.

4-(4''-Methyl)anilino-2-methylthio-5-phenylpyrimidine (42). From *p*-toluidine, yield 81%; mp: 84 °C. ^{1}H NMR (300 MHz, DMSO-d_6): δ 8.35 (s, 1H, NH), 7.99 (s, 1H, 6-H), 7.60-7.45 (m, 5H, Ar-H), 7.44 (d, J = 8.3 Hz, 2H, 2'-H and 6'-H), 7.10 (d, J = 8.4 Hz, 2H, 3'-H and 5'-H), 2.43 (s, 3H, SCH_3), 2.26 (s, 3H, CH_3). Anal. calcd. for $C_{18}H_{17}N_3S$: C, 70.33; H, 5.57; N, 13.67; S, 10.43; found: C, 70.34; H, 5.59; N, 13.70; S, 10.41.

4-(4''-Hydroxy)anilino-2-methylthio-5-phenylpyrimidine (43). From 4-aminophenol, yield 95%; mp: 107 °C. ^{1}H NMR (300 MHz, DMSO-d_6): δ 9.22 (broad s, 1H, OH), 8.21 (s, 1H, NH), 7.93 (s, 1H, 6-H), 7.54-7.39 (m, 5H, Ar-H), 7.30-7.24 (m, 2H, 2''-H and 6''-H), 6.71-6.66 (m,

2H, 3''-H and 5''-H), 2.39 (s, 3H, CH_3). Anal. calcd. for $C_{17}H_{15}N_3OS$: C, 66.00; H, 4.89; N, 13.58; S, 10.36; found: C, 66.03; H, 4.85; N, 13.60; S, 10.37.

4-(4''-Acetamido)anilino-2-methylthio-5-phenylpyrimidine (44). From 4-aminoacetanilide, yield 95%; mp: 173 °C. ^{1}H NMR (300 MHz, DMSO-d_6): δ 9.88 (s, 1H, NH), 8.40 (s, 1H, NH), 7.98 (s, 1H, 6-H), 7.52-7.41 (m, 9H, Ar-H), 2.42 (s, 3H, S-CH_3), 2.02 (s, 3H, CH_3). Anal. calcd. for $C_{19}H_{18}N_4OS$: C, 65.12; H, 5.18; N, 15.99; S, 9.15; found: C, 65.15; H, 5.21; N, 15.96; S, 9.13.

General procedure for 4-anilino-5-phenylpyrimidines 20-25. To a solution of 2-methylthiopyrimidine (**39-44**) (1.0 mmol) in EtOH (20 mL) an aqueous suspension of Raney-Nickel (1.5 mL) was added and the mixture was heated at reflux for 2 h. The mixture was evaporated under reduced pressure to give **20-26** (purified as specified for the individual compounds).

4-(3''-Methyl)anilino-5-phenylpyrimidine (20). From **39**, yield 48% as an oil (purification by flash chromatography, eluting with cyclohexane/EtOAc, 7/3). ^{1}H NMR (300 MHz, DMSO-d_6): δ 8.56 (s, 1H, 6-H), 8.28 (s, 1H, NH), 8.17 (s, 1H 2-H), 7.53-7.45 (m, 5H, Ar-H), 7.41 (d, J = 8.0 Hz, 1H, 6'-H), 7.35 (s, 1H, 2'-H), 7.16 (t, J = 8.0 Hz, 1H, 5'-H), 6.86 (d, J = 8.0 Hz, 1H, 4'-H), 2.26 (s, 3H, CH_3). ^{13}C NMR (75 MHz, DMSO-d_6): δ 157.84, 157.16, 155.56, 139.57, 137.86, 134.56, 129.67, 129.37, 128.73, 128.56, 124.34, 123.07, 120.85, 119.73, 21.54. Anal. calcd. for $C_{17}H_{15}N_3$: C, 78.14; H, 5.79; N, 16.08; found: C, 78.16; H, 5.80; N, 16.06. HRMS (ESI-TOF) for $C_{17}H_{16}N_3$ $[M + H]^+$: calcd, 262.1339; found, 262.1356.

4-(3''-Biphenyl)amino-5-phenylpyrimidine (21). From **40**, yield 33% (purification by flash chromatography, eluting with $CHCl_3$/MeOH, 99/1); mp: 59 °C. ^{1}H NMR (300 MHz, DMSO-d_6): δ 8.59 (s, 1H, 6-H), 8.50 (s, 1H, NH), 8.20 (s, 1H, 2-H), 7.83 (t, J = 1.8 Hz, 1H, 2'-H), 7.70 (d,

J = 8.2 Hz, 1H, Ar-H), 7.64-7.61 (m, 2H, Ar-H), 7.58-7.52 (m, 4H, Ar-H), 7.49-7.44 (m, 3H, Ar-H), 7.41-7.32 (m, 3H, Ar-H). ^{13}C NMR (75 MHz, DMSO-d_6): δ 157.27, 156.57, 155.17, 140.19, 140.04, 139.67, 133.94, 129.11, 128.85, 128.77, 128.67, 128.20, 127.34, 126.49, 121.32, 121.08, 120.41, 120.39. Anal. calcd. for $C_{22}H_{17}N_3$: C, 81.71; H, 5.30; N, 12.99; found: C, 81.71; H, 5.33; N, 12.97. HRMS (ESI-TOF) for $C_{22}H_{18}N_3$ $[M + H]^+$: calcd, 324.1495; found, 324.1485.

4-(3''-Hydroxy)anilino-5-phenylpyrimidine (22). From **41**, yield 32% (crystallization from MeOH); mp: 219 °C. ^{1}H NMR (300 MHz, DMSO-d_6): δ 9.31 (broad s, 1H, OH), 8.58 (s, 1H, NH), 8.21 (s, 1H, 2-H), 8.17 (s, 1H, 6-H), 7.55-7.43 (m, 5H, Ar-H), 7.14-7.10 (m, 1H, 2''-H), 7.04 (t, *J* = 8.0 Hz, 1H, 5''-H), 6.97-6.93 (m, 1H, 6''-H), 6.47-6.42 (m, 1H, 4''-H). ^{13}C NMR (75 MHz, DMSO-d_6): δ 157.22, 157.19, 156.53, 154.98, 140.13, 133.98, 129.11, 128.77, 128.17, 120.38, 112.57, 110.18, 108.90. Anal. calcd. for $C_{16}H_{13}N_3O$: C, 72.99; H, 4.98; N, 15.96; found: C, 72.98; H, 4.99; N, 15.99. HRMS (ESI-TOF) for $C_{16}H_{14}N_3O$ $[M + H]^+$: calcd, 264.1131; found, 264.1086.

4-(4''-Methyl)anilino-5-phenylpyrimidine (23). From **42**, yield 30% (crystallization from *n*-hexane); mp: 138 °C. ^{1}H NMR (300 MHz, DMSO-d_6): δ 8.53 (s, 1H, 2-H), 8.29 (s, 1H, NH), 8.14 (s, 1H, 6-H), 7.55-7.45 (m, 5H, Ar-H), 7.43 (d, *J* = 8.4 Hz, 2H, 2'-H and 6'-H or 3'-H and 5'-H), 7.09 (d, *J* = 8.2 Hz, 2H, 2'-H and 6'-H or 3'-H and 5'-H), 2.26 (s, 3H, CH_3). ^{13}C NMR (75 MHz, DMSO-d_6): δ 157.33, 156.56, 154.75, 136.49, 134.08, 132.16, 129.12, 128.86, 128.60, 128.20, 122.37, 120.13, 20.39. Anal. calcd. for $C_{17}H_{15}N_3$: C, 78.14; H, 5.79; N, 16.08; found: C, 78.15; H, 5.82; N, 16.05. HRMS (ESI-TOF) for $C_{17}H_{16}N_3$ $[M + H]^+$: calcd, 262.1339; found, 262.1386.

4-(4''-Hydroxy)anilino-5-phenylpyrimidine (24). From **43**, yield 19% (crystallization from iPrOH/*n*-hexane); mp: 118 °C. ^{1}H NMR (300 MHz, DMSO-d_6): δ 9.22 (s all, 1H, OH), 8.45 (s,

1H, NH), 8.14 (s, 1H, 2-H), 8.07 (s, 1H, 6-H), 7.54-7.40 (m, 5H, Ar-H), 7.29-7.22 (m, 2H, 2''-H and 6''-H); 6.71-6.65 (m, 2H, 3''-H and 5''-H). ^{13}C NMR (75 MHz, DMSO-*d*$_6$): δ 157.69, 156.60, 154.34, 153.76, 134.11, 130.20, 129.07, 128.77, 128.06, 124.75, 119.58, 114.64. Anal. calcd. for $C_{16}H_{13}N_3O$: C, 72.99; H, 4.98; N, 15.96; found: C, 73.01; H, 5.00; N, 15.94. HRMS (ESI-TOF) for $C_{16}H_{14}N_3O$ [M + H]$^+$: calcd, 264.1131; found, 264.1154.

4-(4''-Amino)anilino-5-phenylpyrimidine (25). From **44**, the acetylamino intermediate was obtained, which was suspended in 3M HCl (5 mL) and heated at reflux for 2 h. The mixture was made alkaline with 3M NaOH, then the obtained precipitate was collected by filtration to give **25**, yield 45%; mp: 86 °C. ^{1}H NMR (300 MHz, DMSO-*d*$_6$): 8.42 (s, 1H, 2-H), 8.03 (s, 1H, 6-H), 7.97 (s, 1H, NH), 7.58-7.40 (m, 5H, Ar-H), 7.09 (d, *J* = 8.7 Hz, 2H, 2'-H and 6'-H or 3'-H and 5'-H); 6.50 (d, J = 8.7 Hz, 2H, 2'-H and 6'-H or 3'-H and 5'-H), 4.91 (s, 2H, NH_2). ^{13}C NMR (75 MHz, DMSO-*d*$_6$): δ 157.82, 156.72, 154.11, 145.28, 134.23, 129.09, 128.76, 128.02, 127.54, 124.73, 119.41, 113.51. Anal. calcd. for $C_{16}H_{14}N_4$: C, 73.26; H, 5.38; N, 21.36; found: C, 73.25; H, 5.41; N, 21.37. HRMS (ESI-TOF) for $C_{16}H_{15}N_4$ [M + H]$^+$: calcd, 263.1291; found, 263.1237.

***N*-phenyl-*N*'-[4'-(5''-phenylpyrimidin-4''-ylamino)phenyl]urea (26).** A solution of **25** (0.16 g, 0.6 mmol) in dichloromethane (5 mL) was added dropwise at 0 °C to a solution of phenyl isocyanate (71.5 mg, 0.6 mmol) in dichloromethane (2 mL) and the mixture was stirred at room temperature for 16 h. After cooling, the obtained precipitate was collected by filtration and purified by flash chromatography (eluting with $CHCl_3$/MeOH, 9/1) to give **26,** yield 28%; mp: 210 °C. ^{1}H NMR (300 MHz, DMSO-*d*$_6$): δ 8.67 (s, 1H, NH), 8.64 (s, 1H, NH), 8.55 (s, 1H, 2''-H), 8.30 (s, 1H, NH), 3.13 (s, 1H, 6''-H), 7.55-7.51 (m, 4H, Ar-H), 7.49-7.42 (m, 5H, Ar-H), 7.37 (d, *J* = 8.9 Hz, 2H, Ar-H), 7.27 (td, *J* = 7.4 Hz, 3.9 Hz, 2H, Ar-H), 6.39 (td, *J* = 7.4 Hz, 2.1

Hz, 1H, Ar-H). ^{13}C NMR (75 MHz, DMSO-d_6): δ 157.42, 156.63, 154.75, 152.49, 139.70, 135.32, 134.07, 133.19, 129.12, 128.83, 128.68, 128.14, 123.28, 121.61, 119.93, 118.10, 118.01. Anal. calcd. for $C_{23}H_{19}N_5O$: C, 72.42; H, 5.02; N, 18.36; found: C, 72.39; H, 5.04; N, 18.37. HRMS (ESI-TOF) for $C_{23}H_{20}N_5O$ $[M + H]^+$: calcd, 382.1662; found, 382.1646.

Synthesis of 4-anilino-6-phenylpyrimidine 27.

Scheme S4. Synthesis of compound **27**[a]

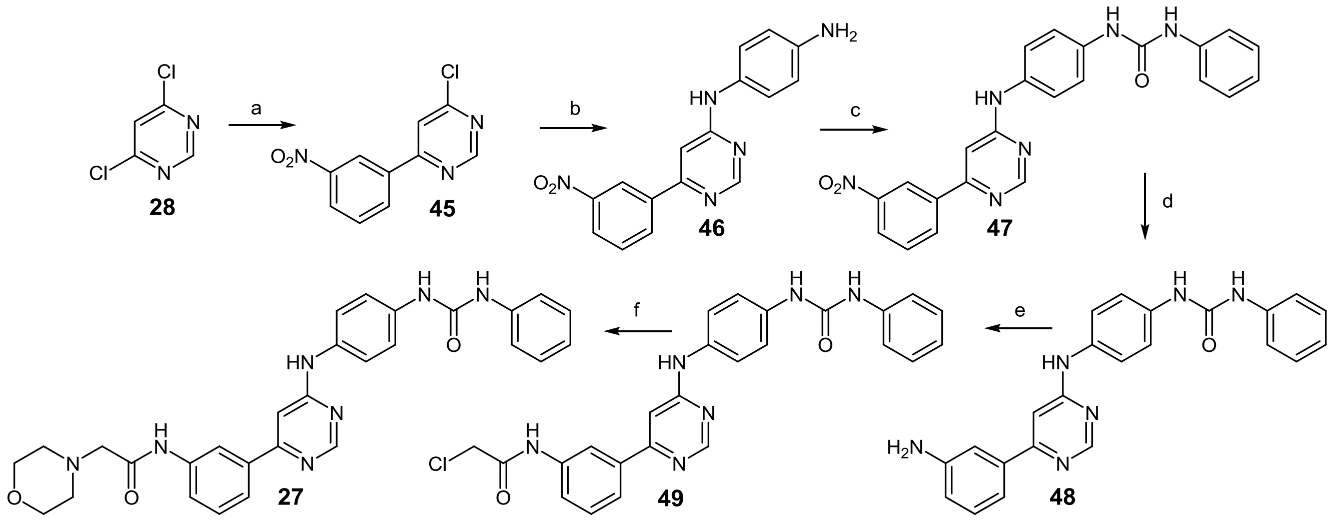

[a]Reagents and conditions: (a) 3-Nitrophenylboronic acid, *tetrakis*(triphenylphosphine)palladium, Na_2CO_3, ethylene glycol dimethyl ether, reflux, 4 h; (b) 1,4-Phenylenediamine, *i*-PrOH, MW, 80 °C, 20 min x 2; (c) Phenyl isocyanate, CH_2Cl_2, RT, 16 h; (d) Ammonium formate, Pd/C, abs. EtOH, reflux, 16 h; (e) Chloroacetyl chloride, TEA, ethylene glycol dimethyl ether, RT, overnight; (f) Morpholine, KI, TEA, DMF, RT, 48 h.

4-Chloro-6-(3-nitrophenyl)pyrimidine (45). To a mixture of **28** (1.5 g, 10 mmol), *tetrakis*(triphenylphosphine)palladium (0.46 g, 0.4 mmol), and 3-nitrophenylboronic acid (0.83 g, 5.0 mmol) in ethylene glycol dimethyl ether (75 mL), a solution of Na_2CO_3 (2.1 g, 20.0 mmol) in water (10 mL) was added. The mixture was heated at reflux for 4 h. After cooling, the solvent was evaporated under reduced pressure and the residue was diluted with sat. NH_4Cl solution (200 mL). The solid was collected by filtration, resuspended in MeOH, then recollected by filtration and purified by flash chromatography (eluting with cyclohexane/AcOEt, 8/2) to give **45** (0.94 g, 78%); mp: 143 °C. ^{1}H NMR (300 MHz, $CDCl_3$-*d*): δ 9.11 (s, 1H, 2-H), 8.95 (t, *J* = 2.0 Hz, 1H, 2'-H), 8.45 (dt, *J* = 7.8, 2.0 Hz, 1H, 4'-H or 6'-H), 8.41 (dt, *J* = 7.8, 2.0 Hz, 1H, 4'-H or 6'-H), 7.85 (s, 1H, 5-H), 7.72 (t, *J* = 7.8 Hz, 1H, 5'-H).

Anal. calcd. for $C_{10}H_6ClN_3O_2$: C, 50.97; H, 2.57; Cl, 15.05; N, 17.83; found: C, 50.99; H, 2.56; Cl, 15.05; N, 17.86.

4-(4'-Amino)anilino-6-(3''-nitro)phenylpyrimidine (46). A mixture of **45** (0.70 g, 3.0 mmol) and 1,4-phenylenediamine (0.49 g, 4.5 mmol) in *i*-PrOH (1.5 mL) was microwave irradiated at 80 °C for three cycles (power set point 80 W; ramp time 1 min; hold time 20 min for each cycle), adding 1,4-phenylenediamine (0.32 g, 3.0 mmol) after the first irradiation cycle. After cooling, the solid was collected by filtration and dissolved in EtOAc (50 mL). The solution was washed with sat. $NaHCO_3$ (3 x 50 mL) and sat. NH_4Cl (3 x 50 mL), then the solvent was evaporated under reduced pressure to give **46** (0.64 g, 70%); mp: 201 °C. ^{1}H NMR (300 MHz, DMSO-d_6): δ 9.31 (s, 1H, NH), 8.78 (t, *J* = 2.4 Hz, 1H, 2''-H), 8.61 (s, 1H, 2-H), 8.39 (dt, *J* = 7.8, 2.4 Hz, 1H, 4''-H or 6''-H), 8.33 (dt, *J* = 7.8, 2.4 Hz, 1H, 4''-H or 6''-H), 7.81 (t, *J* = 7.8 Hz, 1H, 5''-H), 7.23 (d, *J* = 8.4 Hz, 2H, 2'-H and 6'-H), 7.14 (s, 1H, 5-H), 6.58 (d, *J* = 8.4 Hz, 2H, 3'-H and 5'-H), 4.96 (s, 2H, NH_2). Anal. calcd. for $C_{16}H_{13}N_5O_2$: C, 62.53; H, 4.26; N, 22.79; found: C, 62.55; H, 4.26; N, 22.76.

***N*-phenyl-*N*'-{4'-[6''-(3'''-nitrophenyl)pyrimidin-4''-ylamino]phenyl}urea (47).** A solution of **46** (0.44 g, 1.4 mmol) in dichloromethane (8 mL) was added dropwise at 0 °C to a solution of phenyl isocyanate (0.25 g, 2.1 mmol) in dichloromethane (2 mL) and the mixture was stirred at room temperature for 16 h. After cooling, the obtained precipitate was collected by filtration to give **47** (0.50 g, 84%); mp: >300 °C. ^{1}H NMR (300 MHz, DMSO-d_6): δ 9.69 (s, 1H, NH), 8.82 (t, *J* = 2.4 Hz, 1H, 2'''-H), 8.72 (s, 1H, 2''-H), 8.64 (s, 1H, NH), 8.63 (s, 1H, NH), 8.44 (dt, *J* = 7.8, 2.4 Hz, 1H, 4'''-H or 6'''-H), 8.36 (dt, *J* = 7.8, 2.4 Hz, 1H, 4'''-H or 6'''-H), 7.84 (t, *J* = 7.8 Hz, 1H, 5'''-H), 7.61-7.59 (m, 2H, Ar-H), 7.46-7.44 (m, 4H, Ar-H), 7.31 (s, 1H, 5''-H), 7.28-

7.26 (m, 2H, Ar-H), 6.97-6.95 (m, 1H, Ar-H). Anal. calcd. for $C_{23}H_{18}N_6O_3$: C, 64.78; H, 4.25; N, 19.71; found: C, 64.76; H, 4.27; N, 19.70.

***N*-phenyl-*N*'-{4'-[6''-(3'''-aminophenyl)pyrimidin-4''-ylamino]phenyl}urea (48).** A mixture of **47** (0.50 g, 1.2 mmol), ammonium formate (0.37 g, 6.0 mmol) and a catalytic amount of 10% Pd/C in abs. EtOH (100 mL) was stirred under reflux for 16 h. After cooling, the catalyst was filtered off and the solvent was evaporated under reduced pressure to give **48** (0.46 g, 98%); mp: 226 °C. ^{1}H NMR (300 MHz, DMSO-*d*$_6$): δ 9.50 (s, 1H, NH), 8.68 (s, 2H, NH), 8.61 (s, 1H, 2''-H), 7.59-7.56 (m, 2H, Ar-H), 7.47-7.41 (m, 4H, Ar-H), 7.29 (s, 1H, 5''-H), 7.27 (t, *J* = 7.5 Hz, 2H, 3-H and 5-H), 7.14-7.04 (m, 3H, Ar-H), 6.97 (t, *J* = 7.5 Hz, 1H, 4-H), 6.69-6.66 (m, 1H, Ar-H), 5.27 (s, 2H, NH_2). Anal. calcd. for $C_{23}H_{20}N_6O$: C, 69.68; H, 5.08; N, 21.20; found: C, 69.70; H, 5.05; N, 21.23.

***N*-phenyl-*N*'-{{4'-{6''-[3'''-(chloromethylcarbonyl)aminophenyl]pyrimidin-4''-ylamino}-phenyl}}urea (49).** To a solution of **48** (0.40 g, 1.0 mmol) in ethylene glycol dimethyl ether (35 mL), TEA (0.3 mL, 2.0 mmol) and chloroacetyl chloride (0.16 mL, 2.0 mmol) was added and the mixture was stirred at room temperature overnight. The solid was filtered off, the solution was evaporated under reduced pressure and the residue was crystallized from EtOH to give **49** (0.33 g, 70%); mp: >300 °C. ^{1}H NMR (300 MHz, DMSO-*d*$_6$): δ 10.51 (s, 1H, NH), 9.62 (s, 1H, NH), 8.79 (s, 2H, NH), 8.66 (s, 1H, 2''-H), 8.37 (t, *J* = 2.4 Hz, 1H, 2'''-H), 7.74 (dt, *J* = 7.8, 2.4 Hz, 1H, 4'''-H or 6'''-H), 7.69 (dt, *J* = 7.8, 2.4 Hz, 1H, 4'''-H or 6'''-H), 7.61-7.57 (m, 2H, Ar-H), 7.51-7.42 (m, 5H, Ar-H), 7.27 (t, *J* = 7.5 Hz, 2H, 3-H and 5-H), 7.16 (s, 1H, 5''-H), 6.95 (t, *J* = 7.5 Hz, 1H, 4-H), 4.29 (s, 2H, CH_2). Anal. calcd. for $C_{25}H_{21}N_6O_2$: C, 63.49; H, 4.48; Cl, 7.50; N, 17.77; found: C, 63.51; H, 4.49; Cl, 7.53; N, 17.75.

***N*-phenyl-*N'*-{{4'-{6''-[3'''-(morpholinomethylcarbonyl)aminophenyl]pyrimidin-4''-ylamino}-phenyl}}urea (27).** A mixture of **49** (0.33 g, 0.7 mmol), morpholine (86 μL, 1.0 mmol), KI (17 mg, 0.1 mmol) and TEA (0.15 mL, 1.0 mmol) in DMF (6 mL) was stirred at room temperature for 48 h. The mixture was slowly poured in sat. NH_4Cl (60 mL) and the solid was collected by filtration to give **27** (0.3 g, 98%); mp: 285 °C. ^1H NMR (300 MHz, DMSO-d_6): δ 9.92 (s, 1H, NH), 9.60 (s, 1H, NH), 8.66 (s, 1H, 2''-H), 8.61 (s, 2H, NH), 8.40 (broad s, 1H, 2'''-H), 7.71-7.73 (m, 2H, Ar-H), 7.62-7.57 (m, 2H, Ar-H), 7.47-7.42 (m, 5H, Ar-H), 7.27 (t, *J* = 7.5 Hz, 2H, 3-H and 5-H), 7.16 (s, 1H, 5''-H), 6.96 (t, *J* = 7.5 Hz, 1H, 4-H), 3.65 (t, *J* = 4.5 Hz, 4H, OCH_2), 3.17 (s, 2H, $COCH_2$), 2.54 (t, *J* = 4.5 Hz, 4H, NCH_2). ^{13}C NMR (75 MHz, DMSO-d_6): δ 161.31, 154.74, 153.70, 144.59, 139.45, 138.42, 122.74, 118.93, 118.69, 116.79, 116.09, 114.14, 110.68, 107.97, 107.69, 105.95, 105.44, 101.76, 98.12, 95.70, 72.01, 55.20, 49.10. Anal. calcd. for $C_{29}H_{29}N_7O_3$: C, 66.52; H, 5.58; N, 18.73; found: C, 66.49; H, 5.60; N, 18.70. HRMS (ESI-TOF) for $C_{29}H_{30}N_7O_3$ $[M + H]^+$: calcd, 524.2405; found, 524.2381.

Part II: Details on ligands hydration procedure

Before docking simulations, the ".pdbqt" file of compounds **19** and **27** were solvated using the python script (wet.py) described by Forli and Olson.[10]

The coordinates of the water molecule directed to the 3N of pyrimidine (*i.e.* the water molecule supposed to mediate the interaction with the threonine gatekeeper) were slightly modified before docking, according to the distances and angle measured in the crystallographic structure of erlotinib in complex with EGFR (PDB ID: 1M17). The results of the modifications are reported in Figure S1.

Figure S1. Results of ligand hydration procedure on compound 19. Compound **19** is depicted as gray carbon sticks. Water molecules are depicted as cyan sphere. Water molecule directed to the N3 of pyrimidine is highlighted as red sphere. (A) Original results obtained with the wet.py script. (B) Modified structure. (C) Details of crystallographic structure of erlotinib (yellow carbon sticks) in complex with EGFR (PDB ID: 1M17). (D) Dihedral angle between water and pyrimidine as measured in 1M17. Note that the wet.py script place the water molecule on the same plane of the pyrimidine nucleus, whereas in the crystallographic structure of erlotinib it was slightly shifted. ϕ was measured as the angle formed between the plane containing 1,2 and 3 and the vector connecting 3 and 4.

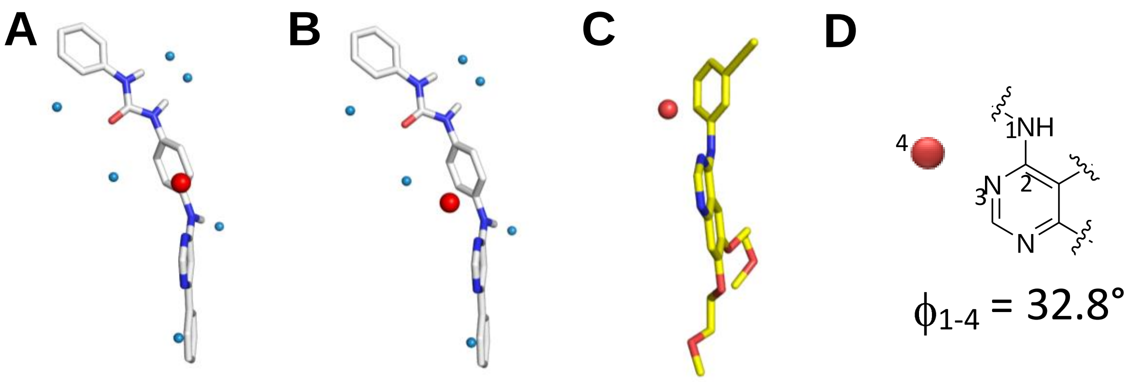

Supplementary Data

Figure S2. Kinase tree. The kinases considered in the present paper are highlighted with red text. Illustration reproduced courtesy of Cell Signaling Technology, Inc. (www.cellsignal.com).

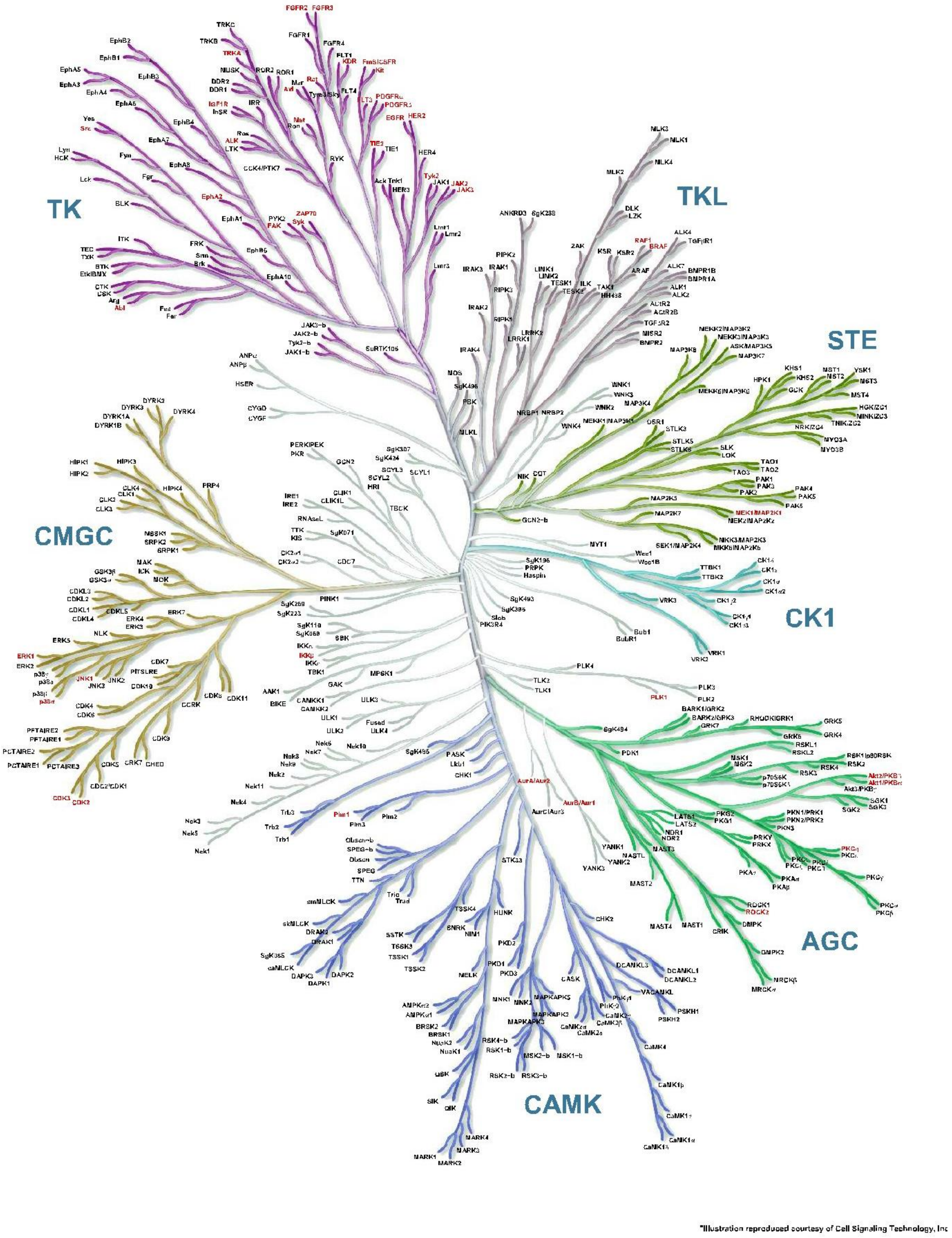

Table S3. List of selected kinases. TK = tyrosine kinase; RTK = receptor tyrosine kinase; TKL = tyrosine kinase-like; MAPK = mitogen-activated protein kinase; STK = serine/threonine kinase; STE = homologs of yeast sterile; CMGC = containing CDK, MAPK, GSK3 and CLK kinases; AGC = protein kinase A, G, and C families; CAMK = Ca^{2+}/calmodulin-dependent protein kinase.

Kinase acronym	Kinase name	Class	Subclass	Ref
ABL1(T315I)-phosphorylated<br>ABL1-nonphosphorylated<br>ABL1-phosphorylated	Abelson tyrosine kinase 1	TK	Cytoplasmic	11
SRC	Src kinase	TK	Cytoplasmic	12
EPHA2	EPH receptor A2	TK	Class VIII RTK	13
FAK	Focal adhesion kinase	TK	Cytoplasmic	14
SYK	Spleen tyrosine kinase	TK	Cytoplasmic	15
ZAP70	70 kDa zeta-associated protein	TK	Cytoplasmic	16
ALK	Anaplastic lymphoma kinase	TK	Class II RTK	17
IGF1R	Insulin growth factor receptor 1	TK	Class II RTK	18
TRKA	Neutrophic tyrosine kinase receptor 1	TK	Class VII RTK	19
AXL	AXL kinase	TK	Class IX RTK	20
MET	Hepatocyte growth factor receptor	TK	Class VI RTK	21
FGFR2	Fibroblast growth factor receptor 2	TK	Class IV RTK	22
FGFR3	Fibroblast growth factor receptor 3	TK	Class IV RTK	23
RET	RET kinase	TK	Class XIV RTK	24
VEGFR2	Vascular endothelial growth factor receptor 2	TK	Class V RTK	25
CSF1R	Colony stimulating factor receptor 1	TK	Class III RTK	26
KIT	Kit kinase	TK	Class III RTK	27
FLT3	Fms-related tyrosine kinase 3	TK	Class III RTK	28
PDGFRA	Platelet-derived growth factor receptor alpha	TK	Class III RTK	29,30
PDGFRB	Platelet-derived growth factor receptor beta	TK	Class III RTK	30
TIE2	TEK tyrosine kinase, endothelial	TK	Class XI RTK	31
EGFR	Epidermal growth factor receptor	TK	Class I RTK	32
EGFR(L858R)	Epidermal growth factor receptor	TK	Class I RTK	33

Table S3. List of selected kinases (continued).

Kinase acronym	Kinase name	Class	Subclass	Ref
EGFR(L858R,T790M)	Epidermal growth factor receptor	TK	Class I RTK	33
ERBB2	Receptor tyrosine kinase erbB-2	TK	Class I RTK	34
TYK2(JH1domain-catalytic)	Tyrosine kinase 2	TK	Cytoplasmic	35
JAK2(JH1domain-catalytic)	Janus kinase 2	TK	Cytoplasmic	36,37
JAK3(JH1domain-catalytic)	Janus kinase 3	TK	Cytoplasmic	37
RAF1	Murine leukemia viral oncogene homolog 1	TKL	MAPK	38
BRAF	Murine sarcoma viral oncogene homolg B1	TKL	MAPK	39
BRAF(V600E)	Murine sarcoma viral oncogene homolg B1	TKL	MAPK	39
MEK1	Mitogen-activated protein kinase 1	STK	STE	40
ERK1	Mitogen-activated protein kinase 3	STK	CMGC	41
p38-alpha	Mitogen-activated protein kinase 14	STK	CMGC	42
JNK1	Mitogen-activated protein kinase 8	STK	CMGC	43
CDK2	Cyclin-dependent kinase 2	STK	CMGC	44
CDK3	Cyclin-dependent kinase 3	STK	CMGC	44
IKK-beta	I-kappa-B kinase 2	STK	Other	45
PLK1	Polo-like kinase 1	STK	Other	46
AURKA	Aurora kinase A	STK	Other	47
AURKB	Aurora kinase B	STK	Other	47
AKT1	Protein kinase B alpha	STK	AGC	48
AKT2	Protein kinase B beta	STK	AGC	49
PRKCH	Protein kinase C eta	STK	AGC	50
ROCK2	Rho associated kinase	STK	AGC	51
PIM1	Pim-1 kinase	STK	CAMK	52

Table S4. Measured POC values.

	1	2	3	4	5	6	7	8	9
ABL1(T315I)-phosphorylated	96	100	86	100	99	86	85	95	77
ABL1-nonphosphorylated	100	90	93	70	100	68	65	89	43
ABL1-phosphorylated	92	94	77	79	95	78	75	81	46
SRC	100	100	92	96	100	100	48	78	30
EPHA2	100	100	86	100	97	35	100	100	100
FAK	100	98	98	100	97	100	99	93	70
SYK	100	100	97	68	97	85	76	89	60
ZAP70	83	63	61	65	70	72	67	65	70
ALK	75	87	71	60	70	61	81	91	58
IGF1R	95	99	94	97	97	31	90	100	80
TRKA	94	89	53	78	96	79	84	77	21
AXL	96	92	75	64	70	90	41	38	6
MET	100	87	59	86	100	76	66	100	42
FGFR2	100	100	100	100	100	100	100	100	76
FGFR3	91	99	80	90	73	89	91	90	62
RET	95	82	69	70	85	87	82	66	37
VEGFR2	100	100	85	91	97	85	90	91	57
CSF1R	85	100	66	90	82	84	14	36	22
KIT	18	57	26	31	70	24	23	38	7
FLT3	18	34	18	21	24	17	10	44	5
PDGFRA	77	83	66	53	100	65	64	68	22
PDGFRB	57	67	39	38	65	35	20	34	11
TIE2	100	97	72	100	88	100	100	100	90
EGFR	55	82	86	75	80	70	1	8	2
EGFR(L858R)	67	73	85	84	75	98	0	5	1
EGFR(L858R,T790M)	100	100	79	84	100	72	78	85	50
ERBB2	88	88	86	81	85	86	64	87	42
TYK2(JH1domain-catalytic)	100	86	73	77	96	76	74	72	44
JAK2(JH1domain-catalytic)	73	77	52	74	71	79	68	75	48
JAK3(JH1domain-catalytic)	75	86	38	75	78	74	89	73	40
RAF1	92	96	94	100	95	79	87	100	91
BRAF	83	100	92	100	97	92	91	83	81
BRAF(V600E)	97	100	88	100	85	100	100	100	80
MEK1	100	94	82	100	82	100	100	92	81
ERK1	93	100	100	100	100	88	100	90	88
p38-alpha	100	100	100	100	100	100	87	84	73
JNK1	92	91	98	86	100	66	91	78	46
CDK2	82	85	74	85	83	91	83	82	64
CDK3	100	100	86	87	97	91	84	96	71
IKK-beta	99	100	88	100	85	80	88	89	65
PLK1	100	100	85	88	83	100	94	84	79
AURKA	95	92	77	90	92	93	71	88	60
AURKB	83	70	64	88	52	82	80	76	51
AKT1	100	100	100	100	91	100	100	100	80
AKT2	100	100	95	88	97	39	88	91	79
PRKCH	100	100	100	100	100	58	100	100	81
ROCK2	100	100	100	100	100	96	82	100	64
PIM1	98	85	76	84	100	85	64	89	72

Table S4 (Continued)

	10	11	12	13	14	15	16	17	18
ABL1(T315I)-phosphorylated	94	90	81	89	80	91	51	87	90
ABL1-nonphosphorylated	97	83	34	66	82	80	27	100	80
ABL1-phosphorylated	88	86	40	62	79	77	30	93	79
SRC	74	74	40	32	66	64	35	78	76
EPHA2	100	100	100	100	100	100	100	100	100
FAK	99	96	85	88	96	100	75	100	100
SYK	93	72	74	76	87	92	57	83	100
ZAP70	68	52	68	78	74	74	71	71	57
ALK	67	63	56	70	62	75	28	66	79
IGF1R	91	89	91	92	89	100	35	100	97
TRKA	72	95	38	66	78	90	4	84	85
AXL	60	60	6	49	72	74	1	100	86
MET	100	74	48	64	80	92	32	74	95
FGFR2	100	99	100	100	100	100	50	100	100
FGFR3	82	75	76	84	80	84	31	85	89
RET	77	76	63	93	83	87	25	86	84
VEGFR2	80	90	70	80	87	100	28	94	96
CSF1R	93	77	16	9	71	71	13	95	90
KIT	81	64	8	34	78	66	1	90	75
FLT3	68	53	2	44	67	61	1	75	55
PDGFRA	90	85	19	74	81	75	10	91	93
PDGFRB	62	77	9	36	81	35	4	100	55
TIE2	100	100	96	96	96	84	100	100	100
EGFR	2	6	1	0	0	12	2	17	24
EGFR(L858R)	1	7	1	0	0	13	2	43	30
EGFR(L858R,T790M)	100	89	28	76	75	100	14	99	85
ERBB2	73	91	29	64	76	87	21	94	39
TYK2(JH1domain-catalytic)	76	82	55	60	77	81	23	92	78
JAK2(JH1domain-catalytic)	58	76	53	66	77	76	18	62	66
JAK3(JH1domain-catalytic)	84	75	36	75	79	86	7	92	85
RAF1	69	75	77	96	98	90	100	95	93
BRAF	79	97	81	93	97	87	70	83	85
BRAF(V600E)	85	97	88	100	100	90	100	95	84
MEK1	73	100	91	100	90	100	37	100	98
ERK1	94	94	90	100	96	96	88	92	100
p38-alpha	80	100	67	78	90	97	71	100	100
JNK1	73	100	24	100	97	90	23	98	100
CDK2	76	81	76	82	85	70	47	83	85
CDK3	96	100	96	82	80	92	72	84	100
IKK-beta	99	68	78	87	92	82	56	83	100
PLK1	95	84	87	90	100	94	46	81	100
AURKA	81	89	55	92	89	100	25	83	88
AURKB	68	78	56	79	84	82	38	63	74
AKT1	100	92	100	100	90	87	100	83	100
AKT2	97	100	91	91	96	100	86	80	97
PRKCH	100	17	81	93	94	98	71	94	91
ROCK2	96	67	71	84	100	92	19	100	100
PIM1	85	89	87	81	77	96	63	87	70

Table S4 (Continued)

	19	20	21	22	23	24	25	26
ABL1(T315I)-phosphorylated	91	100	93	15	89	99	98	100
ABL1-nonphosphorylated	89	72	80	8	82	82	89	100
ABL1-phosphorylated	93	82	90	16	93	82	92	97
SRC	79	77	85	83	98	100	79	99
EPHA2	100	100	100	100	100	100	100	100
FAK	88	98	98	95	94	100	100	100
SYK	66	95	97	86	100	100	73	98
ZAP70	66	73	69	67	64	50	72	65
ALK	82	77	65	74	79	78	97	81
IGF1R	92	96	98	87	100	94	100	100
TRKA	74	76	73	82	68	48	58	74
AXL	82	70	89	58	76	100	40	67
MET	87	78	71	73	89	65	65	84
FGFR2	100	100	100	100	100	100	100	100
FGFR3	76	90	85	81	86	87	86	98
RET	77	62	60	78	63	79	51	37
VEGFR2	89	95	100	100	91	71	70	5
CSF1R	90	0	72	75	78	4	1	0
KIT	78	15	39	24	28	0	0	0
FLT3	65	14	48	62	4	20	0	0
PDGFRA	85	45	66	87	54	9	16	2
PDGFRB	73	19	45	58	28	0	0	0
TIE2	97	100	93	82	94	100	100	88
EGFR	34	35	26	12	55	87	13	58
EGFR(L858R)	42	45	32	14	54	100	10	57
EGFR(L858R,T790M)	99	76	100	50	86	92	80	87
ERBB2	63	52	43	12	82	100	94	100
TYK2(JH1domain-catalytic)	79	85	78	79	73	85	74	79
JAK2(JH1domain-catalytic)	77	72	70	86	76	61	85	70
JAK3(JH1domain-catalytic)	99	78	100	81	88	86	82	93
RAF1	100	98	100	100	94	93	98	100
BRAF	83	89	86	100	98	85	86	91
BRAF(V600E)	92	93	91	100	78	100	94	83
MEK1	94	83	66	100	98	100	95	63
ERK1	98	97	100	74	94	100	92	96
p38-alpha	88	100	93	96	100	100	93	100
JNK1	100	99	100	100	100	80	65	100
CDK2	94	92	92	78	89	89	82	73
CDK3	85	92	94	86	91	76	73	100
IKK-beta	84	76	100	78	86	86	59	100
PLK1	81	96	94	92	88	90	100	100
AURKA	76	88	81	95	86	61	87	54
AURKB	68	77	84	5	79	66	65	38
AKT1	93	96	98	90	100	100	100	100
AKT2	94	89	93	94	100	100	100	100
PRKCH	100	44	26	94	92	100	90	93
ROCK2	98	82	57	88	94	89	95	28
PIM1	82	87	84	95	78	90	84	86

Figure S3. Dose/response curve for compound 19 against tested kinases for Kd determination. The amount of kinase measured by qPCR (Signal; y-axis) is plotted against the corresponding compound concentration in nM in log10 scale (x-axis). Data points marked with an "x" were not used for Kd determination.

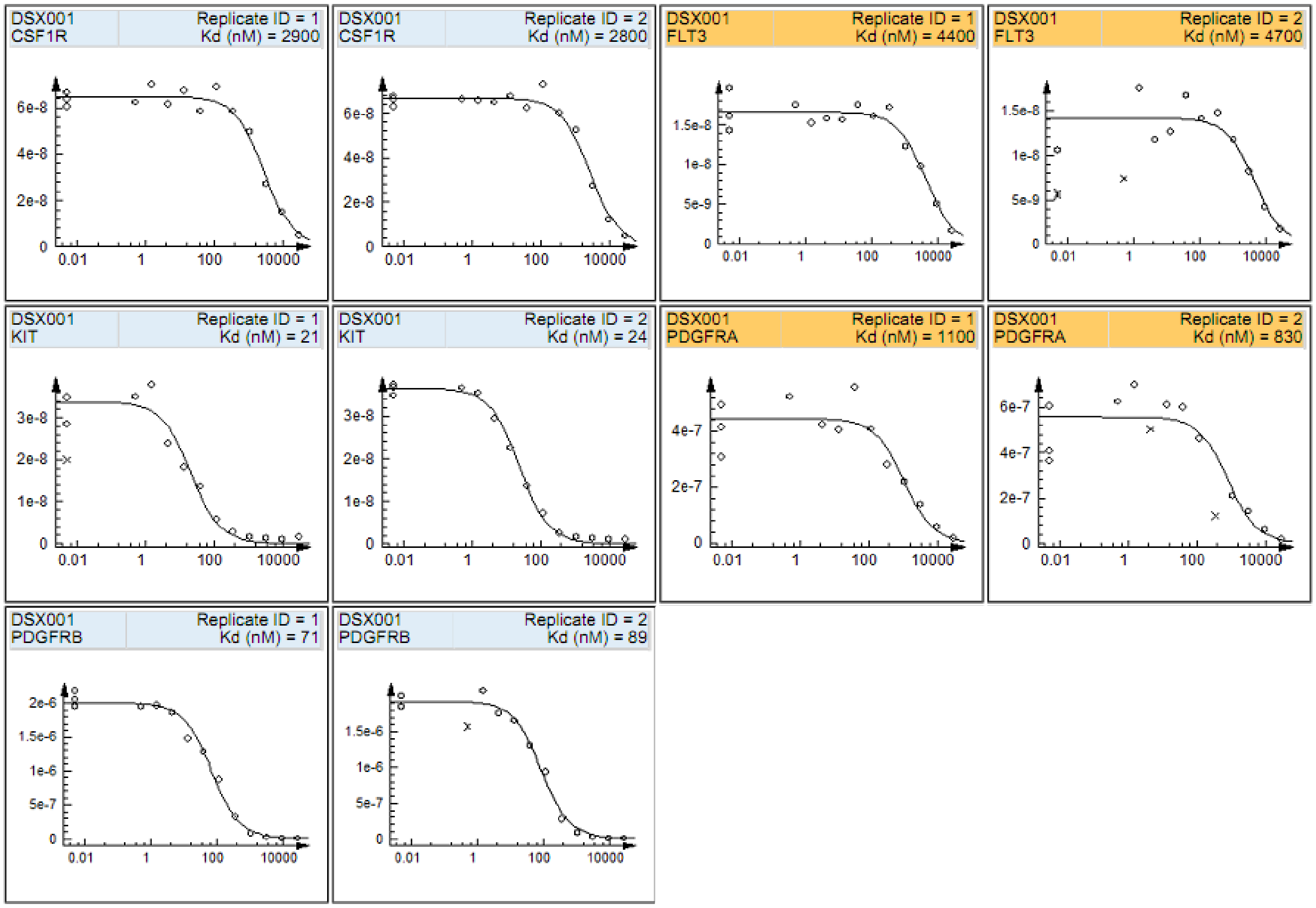

Figure S4. Dose/response curve for compound 27 against tested kinases for Kd determination. The amount of kinase measured by qPCR (Signal; y-axis) is plotted against the corresponding compound concentration in nM in log10 scale (x-axis). Data points marked with an "x" were not used for Kd determination.

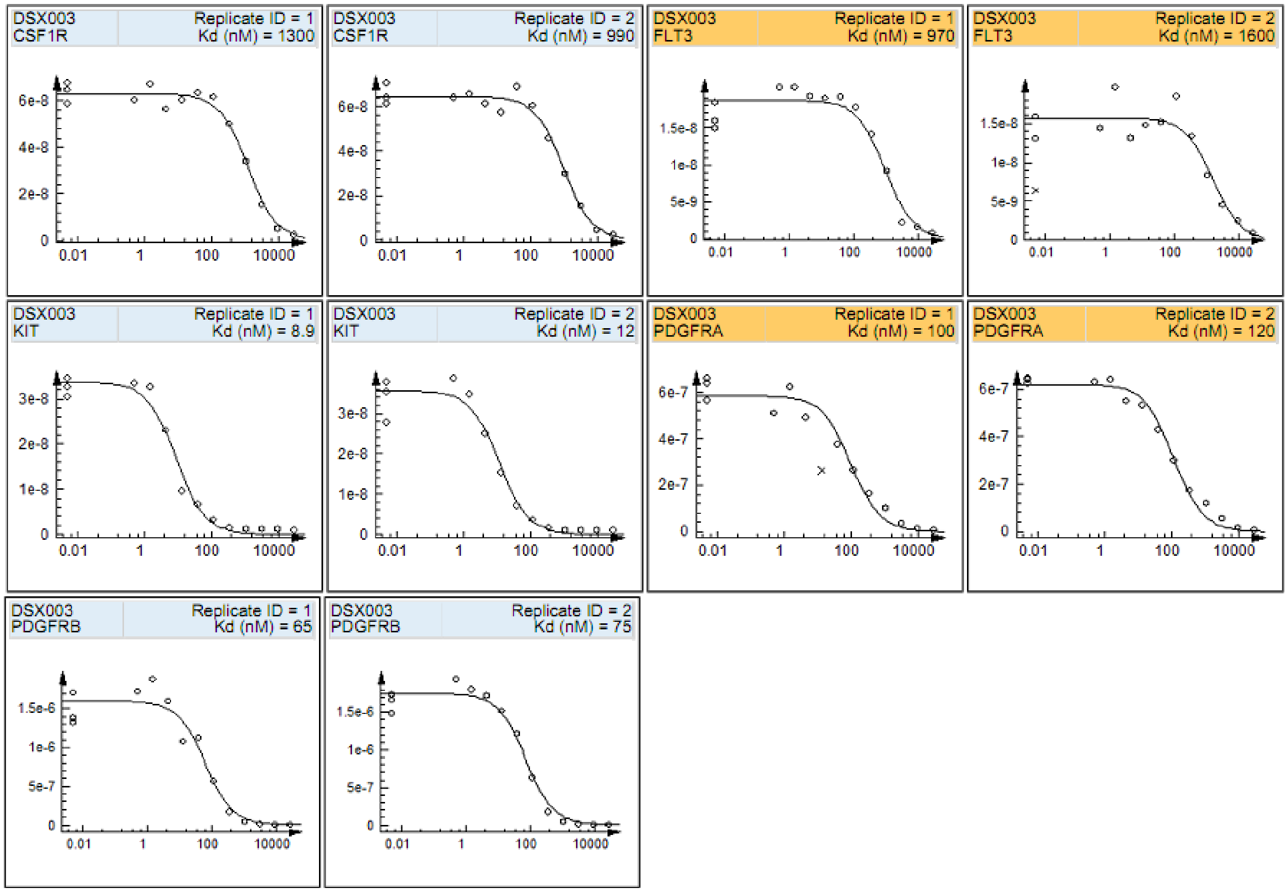

Table S5. Results of in vivo testing for compound 27. Lewis lung carcinoma (LLC) was implanted i.m. ($2 \cdot 10^6$ cells inoculum) into the right hind leg of 8-week old imbred C57BL mice. Nine days after tumor inoculation (palpable tumor), tumor-bearing mice were randomized into vehicle control and treatment groups (8 mice per group). Compound 49 was dosed daily at 7.5 mg/kg ip and CDDP was dosed daily at 1.5 mg/kg ip. At day 20, animals were sacrificed (*i.e.*, before tumor can cause the animal discomfort), the legs were amputated at the proximal end of the femur, and the inhibition of tumor growth was determined according to the difference in weight of the tumor-bearing leg and the healthy leg of the animals expressed as % referred to the control animals. Control is constituted by vehicle (0.2 % EtOH (v/v) and 99.8% of saline solution (v/v))

	**Daily dose ($mg \cdot kg^{-1}$)**	**Average tumor weight (mean±S.D., g)**	**Inhibition of tumor growth (%)**
**control**	-	0.490±0.13	-
**27**	7.5	0.075±0.03	84.70
**CDDP**	1.5	0.139±0.09	71.63

Figure S5. Details of proposed binding mode of 19 in KIT. The numbers on the right panel indicate the distances between donor and acceptor (in the cases of H-bonds) or the distance between the center of aromatic rings (in the case of T-shaped arene/arene interactions).

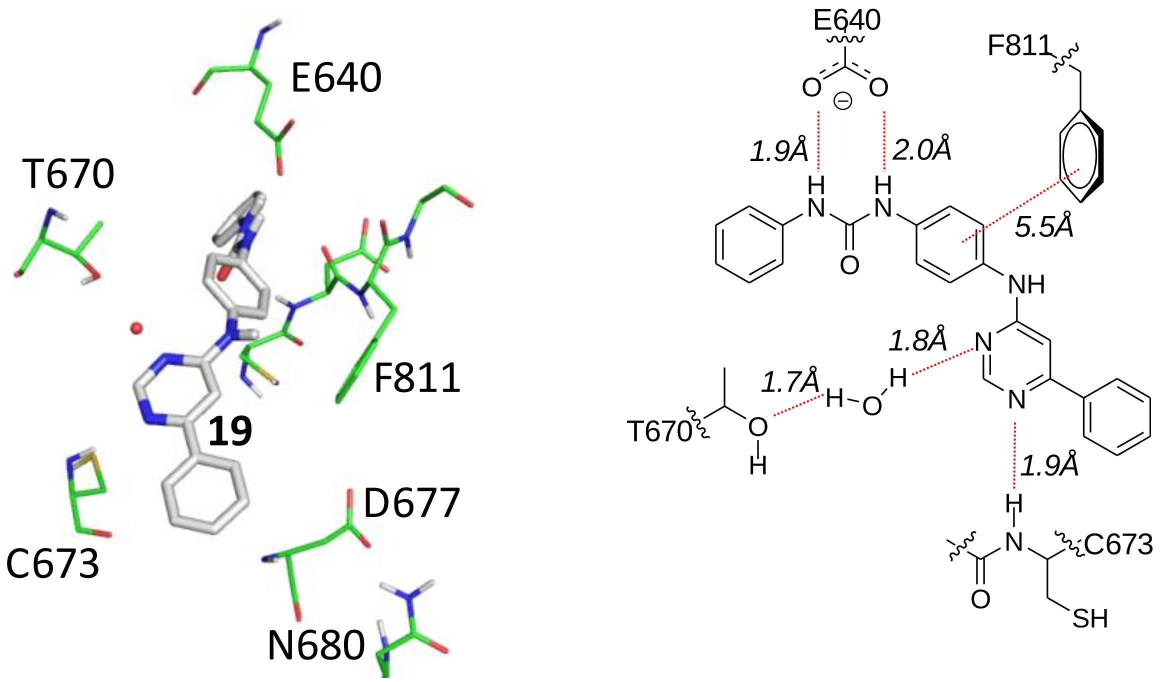

Figure S6. Details of proposed binding mode of 27 in KIT. The numbers on the right panel indicate the distances between donor and acceptor (in the cases of H-bonds) or the distance between the center of aromatic rings (in the case of T-shaped arene/arene interactions).

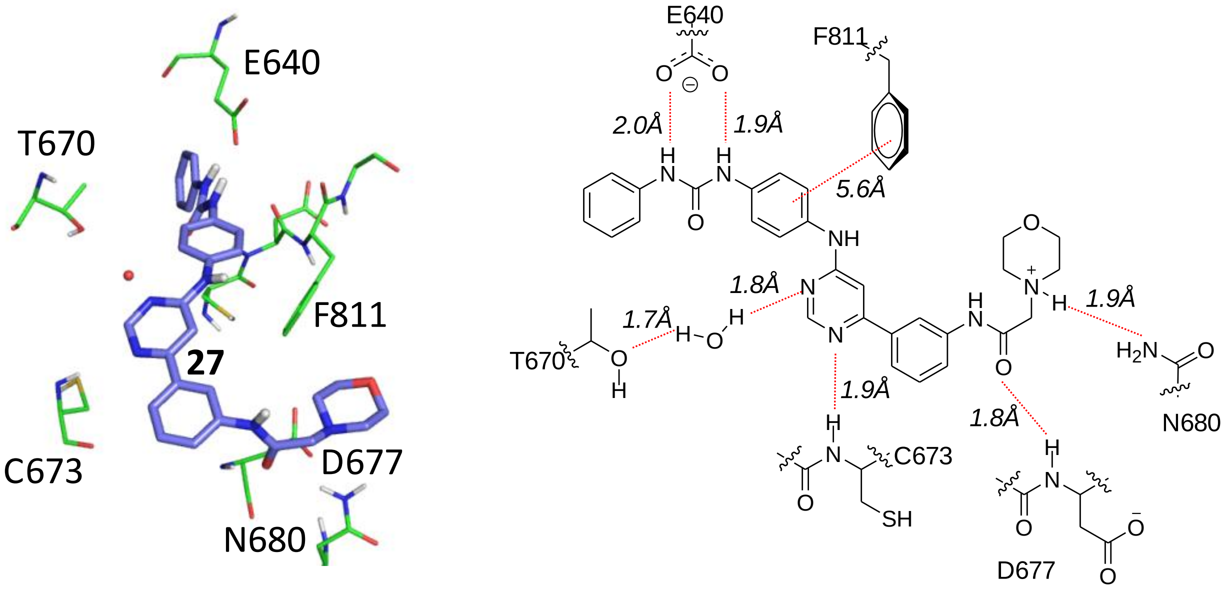

Figure S7. Details of proposed binding mode of 19 in CSF1R. The numbers on the right panel indicate the distances between donor and acceptor (in the cases of H-bonds) or the distance between the center of aromatic rings (in the case of T-shaped arene/arene interactions).

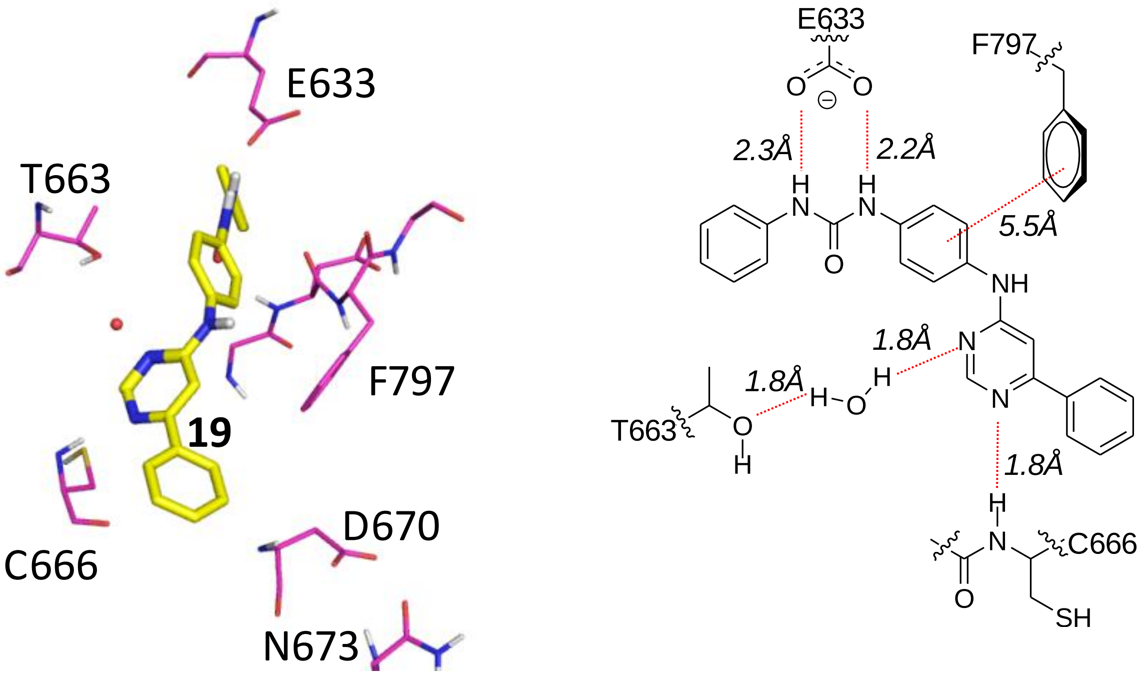

Figure S8. Details of proposed binding mode of 19 in FLT3. The numbers on the right panel indicate the distances between donor and acceptor (in the cases of H-bonds) or the distance between the center of aromatic rings (in the case of T-shaped arene/arene interactions).

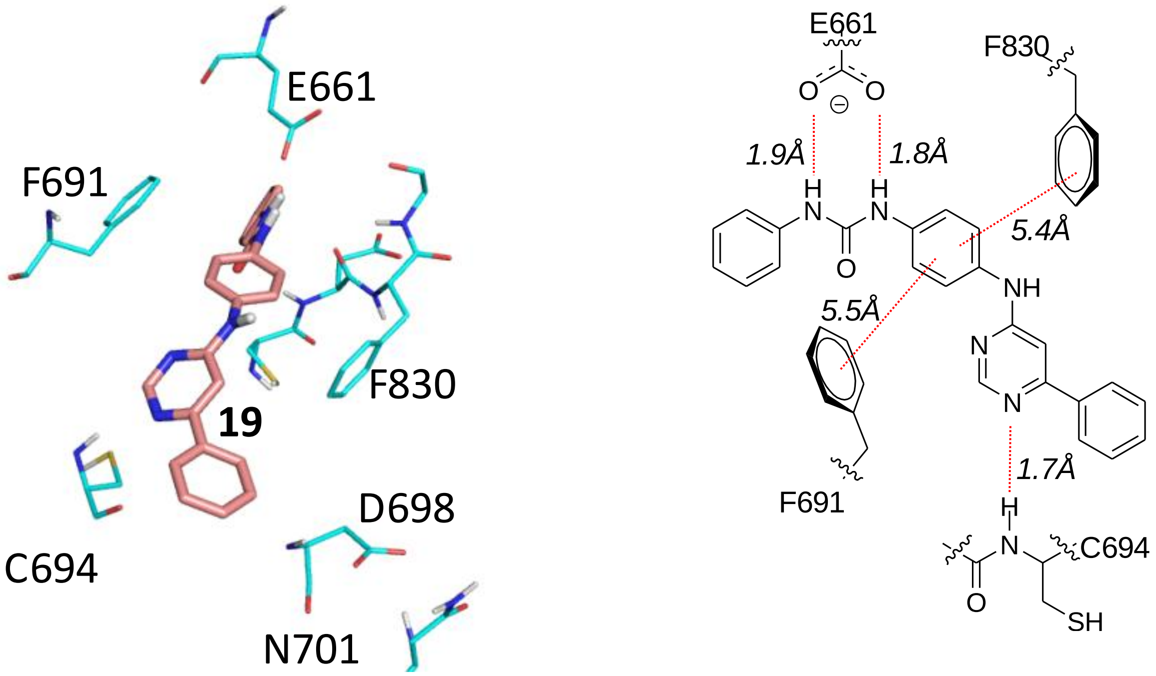

Supplementary References

1 Beattie, J. F. *et al.* Cyclin-dependent kinase 4 inhibitors as a treatment for cancer. Part 1: identification and optimisation of substituted 4,6-bis anilino pyrimidines. *Bioorg Med Chem Lett* **13**, 2955-2960 (2003).

2 Zhang, Q. *et al.* Discovery of EGFR selective 4,6-disubstituted pyrimidines from a combinatorial kinase-directed heterocycle library. *J Am Chem Soc* **128**, 2182-2183 (2006).

3 Gozalbes, R. *et al.* Development and experimental validation of a docking strategy for the generation of kinase-targeted libraries. *J Med Chem* **51**, 3124-3132 (2008).

4 Deng, X. *et al.* Expanding the diversity of allosteric bcr-abl inhibitors. *J Med Chem* **53**, 6934-6946 (2010).

5 Jahnke, W. *et al.* Binding or bending: distinction of allosteric Abl kinase agonists from antagonists by an NMR-based conformational assay. *J Am Chem Soc* **132**, 7043-7048 (2010).

6 Schneider, R. *et al.* Direct binding assay for the detection of type IV allosteric inhibitors of Abl. *J Am Chem Soc* **134**, 9138-9141 (2012).

7 Albert, T. K. *et al.* Characterization of molecular and cellular functions of the cyclin-dependent kinase CDK9 using a novel specific inhibitor. *Br J Pharmacol* **171**, 55-68 (2012).

8 Park, H., Shin, Y., Choe, H. & Hong, S. Computational design and discovery of nanomolar inhibitors of IkappaB kinase beta. *J Am Chem Soc* **137**, 337-348 (2015).

9 Cha, M. Y. *et al.* Synthesis and biological evaluation of pyrimidine-based dual inhibitors of human epidermal growth factor receptor 1 (HER-1) and HER-2 tyrosine kinases. *J Med Chem* **55**, 2846-2857 (2012).

10 Forli, S. & Olson, A. J. A force field with discrete displaceable waters and desolvation entropy for hydrated ligand docking. *J Med Chem* **55**, 623-638 (2012).

11 Greuber, E. K., Smith-Pearson, P., Wang, J. & Pendergast, A. M. Role of ABL family kinases in cancer: from leukaemia to solid tumours. *Nat Rev Cancer* **13**, 559-571 (2013).

12 Guarino, M. Src signaling in cancer invasion. *J Cell Physiol* **223**, 14-26 (2010).

13 Dunne, P. D. *et al.* EphA2 expression is a key driver of migration and invasion and a poor prognostic marker in colorectal cancer. *Clin Cancer Res* (2015).

14 Sulzmaier, F. J., Jean, C. & Schlaepfer, D. D. FAK in cancer: mechanistic findings and clinical applications. *Nat Rev Cancer* **14**, 598-610 (2014).

15 Ghotra, V. P. *et al.* SYK is a candidate kinase target for the treatment of advanced prostate cancer. *Cancer Res* **75**, 230-240 (2015).

16 Zhao, H. & Caflisch, A. Discovery of ZAP70 inhibitors by high-throughput docking into a conformation of its kinase domain generated by molecular dynamics. *Bioorg Med Chem Lett* **23**, 5721-5726 (2013).

17 Croegaert, K. & Kolesar, J. M. Role of anaplastic lymphoma kinase inhibition in the treatment of non-small-cell lung cancer. *Am J Health Syst Pharm* **72**, 1456-1462 (2015).

18 Larsson, O., Girnita, A. & Girnita, L. Role of insulin-like growth factor 1 receptor signalling in cancer. *Br J Cancer* **96 Suppl**, R2-6 (2007).

19 Lagadec, C. *et al.* TrkA overexpression enhances growth and metastasis of breast cancer cells. *Oncogene* **28**, 1960-1970 (2009).

20 Paccez, J. D., Vogelsang, M., Parker, M. I. & Zerbini, L. F. The receptor tyrosine kinase Axl in cancer: biological functions and therapeutic implications. *Int J Cancer* **134**, 1024-1033 (2014).

21 Gherardi, E., Birchmeier, W., Birchmeier, C. & Vande Woude, G. Targeting MET in cancer: rationale and progress. *Nat Rev Cancer* **12**, 89-103 (2012).

22 Reintjes, N. *et al.* Activating somatic FGFR2 mutations in breast cancer. *PLoS One* **8**, e60264 (2013).

23 Acquaviva, J. *et al.* FGFR3 translocations in bladder cancer: differential sensitivity to HSP90 inhibition based on drug metabolism. *Mol Cancer Res* **12**, 1042-1054 (2014).

24 Mulligan, L. M. RET revisited: expanding the oncogenic portfolio. *Nat Rev Cancer* **14**, 173-186 (2014).

25 Paz, K. & Zhu, Z. Development of angiogenesis inhibitors to vascular endothelial growth factor receptor 2. Current status and future perspective. *Front Biosci* **10**, 1415-1439 (2005).

26 Patel, S. & Player, M. R. Colony-stimulating factor-1 receptor inhibitors for the treatment of cancer and inflammatory disease. *Curr Top Med Chem* **9**, 599-610 (2009).

27 Ashman, L. K. & Griffith, R. Therapeutic targeting of c-KIT in cancer. *Expert Opin Investig Drugs* **22**, 103-115 (2013).

28 Stirewalt, D. L. & Radich, J. P. The role of FLT3 in haematopoietic malignancies. *Nat Rev Cancer* **3**, 650-665 (2003).

29 Cenciarelli, C. *et al.* PDGF receptor alpha inhibition induces apoptosis in glioblastoma cancer stem cells refractory to anti-Notch and anti-EGFR treatment. *Mol Cancer* **13**, 247 (2014).

30 Hofer, M. D. *et al.* Expression of the platelet-derived growth factor receptor in prostate cancer and treatment implications with tyrosine kinase inhibitors. *Neoplasia* **6**, 503-512 (2004).

31 Huang, H., Bhat, A., Woodnutt, G. & Lappe, R. Targeting the ANGPT-TIE2 pathway in malignancy. *Nat Rev Cancer* **10**, 575-585 (2010).

32 Normanno, N. *et al.* Epidermal growth factor receptor (EGFR) signaling in cancer. *Gene* **366**, 2-16 (2006).

33 Gazdar, A. F. Activating and resistance mutations of EGFR in non-small-cell lung cancer: role in clinical response to EGFR tyrosine kinase inhibitors. *Oncogene* **28 Suppl 1**, S24-31 (2009).

34 Yu, D. & Hung, M. C. Overexpression of ErbB2 in cancer and ErbB2-targeting strategies. *Oncogene* **19**, 6115-6121 (2000).

35 Zhang, Q. *et al.* The role of Tyk2 in regulation of breast cancer growth. *J Interferon Cytokine Res* **31**, 671-677 (2011).

36 Miller, C. P. *et al.* JAK2 expression is associated with tumor-infiltrating lymphocytes and improved breast cancer outcomes: implications for evaluating JAK2 inhibitors. *Cancer Immunol Res* **2**, 301-306 (2014).

37 Kontzias, A., Kotlyar, A., Laurence, A., Changelian, P. & O'Shea, J. J. Jakinibs: a new class of kinase inhibitors in cancer and autoimmune disease. *Curr Opin Pharmacol* **12**, 464-470 (2012).

38 McPhillips, F. *et al.* Raf-1 is the predominant Raf isoform that mediates growth factor-stimulated growth in ovarian cancer cells. *Carcinogenesis* **27**, 729-739 (2006).

39 Davies, H. *et al.* Mutations of the BRAF gene in human cancer. *Nature* **417**, 949-954 (2002).

40 Sogabe, S. *et al.* MEK inhibitor for gastric cancer with MEK1 gene mutations. *Mol Cancer Ther* **13**, 3098-3106 (2014).

41 Meloche, S. & Pouyssegur, J. The ERK1//2 mitogen-activated protein kinase pathway as a master regulator of the G1- to S-phase transition. *Oncogene* **26**, 3227-3239 (2007).

42 Gupta, J. *et al.* Dual function of p38alpha MAPK in colon cancer: suppression of colitis-associated tumor initiation but requirement for cancer cell survival. *Cancer Cell* **25**, 484-500 (2014).

43 Bubici, C. & Papa, S. JNK signalling in cancer: in need of new, smarter therapeutic targets. *Br J Pharmacol* **171**, 24-37 (2014).

44 Shapiro, G. I. Cyclin-dependent kinase pathways as targets for cancer treatment. *J Clin Oncol* **24**, 1770-1783 (2006).

45 Jiang, R. *et al.* High expression levels of IKKalpha and IKKbeta are necessary for the malignant properties of liver cancer. *Int J Cancer* **126**, 1263-1274 (2010).

46 Strebhardt, K. & Ullrich, A. Targeting polo-like kinase 1 for cancer therapy. *Nat Rev Cancer* **6**, 321-330 (2006).

47 Keen, N. & Taylor, S. Aurora-kinase inhibitors as anticancer agents. *Nat Rev Cancer* **4**, 927-936 (2004).

48 Davies, M. A. Regulation, role, and targeting of Akt in cancer. *J Clin Oncol* **29**, 4715-4717 (2011).

49 Rychahou, P. G. *et al.* Akt2 overexpression plays a critical role in the establishment of colorectal cancer metastasis. *Proc Natl Acad Sci U S A* **105**, 20315-20320 (2008).

50 Brenner, W. *et al.* Protein kinase C eta is associated with progression of renal cell carcinoma (RCC). *Anticancer Res* **23**, 4001-4006 (2003).

51 Vigil, D. *et al.* ROCK1 and ROCK2 are required for non-small cell lung cancer anchorage-independent growth and invasion. *Cancer Res* **72**, 5338-5347 (2012).

52 Shah, N. *et al.* Potential roles for the PIM1 kinase in human cancer - a molecular and therapeutic appraisal. *Eur J Cancer* **44**, 2144-2151 (2008).
